# Priming of transcriptional memory responses via the chromatin accessibility landscape in T cells

**DOI:** 10.1038/srep44825

**Published:** 2017-03-20

**Authors:** Wen Juan Tu, Kristine Hardy, Christopher R. Sutton, Robert McCuaig, Jasmine Li, Jenny Dunn, Abel Tan, Vedran Brezar, Melanie Morris, Gareth Denyer, Sau Kuen Lee, Stephen J. Turner, Nabila Seddiki, Corey Smith, Rajiv Khanna, Sudha Rao

**Affiliations:** 1 Faculty of Education, Science, Technology & Mathematics, University of Canberra, Canberra, Australian Capital Territory 2617, Australia; 2 Department of Microbiology, Biomedical Discovery Institute, Monash University, Clayton, Victoria 3800, Australia; 3 Department of Microbiology & Immunology, The Doherty Institute for Infection and Immunity, University of Melbourne, Victoria 3010, Australia; 4 INSERM U955 Eq16 Faculte de medicine Henri Mondor and Universite Paris-Est, Creteil/Vaccine Research Institute, Creteil 94010, France; 5 School of Molecular Bioscience, The University of Sydney, Sydney, NSW, Australia; 6 QIMR Berghofer Centre for Immunotherapy and Vaccine Development QIMR Berghofer Medical Research Institute, Brisbane, Queensland, Australia; 7 Tumour Immunology Laboratory, Department of Immunology, QIMR Berghofer Medical Research Institute, Brisbane, Queensland, Australia

## Abstract

Memory T cells exhibit transcriptional memory and “remember” their previous pathogenic encounter to increase transcription on re-infection. However, how this transcriptional priming response is regulated is unknown. Here we performed global FAIRE-seq profiling of chromatin accessibility in a human T cell transcriptional memory model. Primary activation induced persistent accessibility changes, and secondary activation induced secondary-specific opening of previously less accessible regions associated with enhanced expression of memory-responsive genes. Increased accessibility occurred largely in distal regulatory regions and was associated with increased histone acetylation and relative H3.3 deposition. The enhanced re-stimulation response was linked to the strength of initial PKC-induced signalling, and PKC-sensitive increases in accessibility upon initial stimulation showed higher accessibility on re-stimulation. While accessibility maintenance was associated with ETS-1, accessibility at re-stimulation-specific regions was linked to NFAT, especially in combination with ETS-1, EGR, GATA, NFκB, and NR4A. Furthermore, *NFATC1* was directly regulated by ETS-1 at an enhancer region. In contrast to the factors that increased accessibility, signalling from bHLH and ZEB family members enhanced decreased accessibility upon re-stimulation. Interplay between distal regulatory elements, accessibility, and the combined action of sequence-specific transcription factors allows transcriptional memory-responsive genes to “remember” their initial environmental encounter.

Naïve T cells exist at rest until exposed to activating signals from antigen presenting cells. This activates transcription to co-ordinate proliferation, differentiation, and the production of inflammatory molecules to clear infection. Naïve and memory T cell transcriptomes are similar apart from a distinct subset of genes involved in processes such as cell adhesion and survival^1^,^2^. In contrast to naïve T cells, memory T cells are primed for a rapid response to antigenic re-exposure^1^,^2^,^3^. This enhanced response is in part attributed to more efficient T cell receptor (TCR) signalling such as increased activity of ZAP-70^4^, the MAP kinases^5^,^6^, and protein kinase C (PKC)^7^. PKC family members -θ, η, α, δ are important in T lymphocyte signalling^8^,^9^. T cell activation with phorbol 12-myristate 13-acetate (PMA) can activate the “novel” PKCs (including PKC-θ) and, when administered with calcium ionophore, the conventional PKCs^10^. Together, PMA and calcium ionophore mimic T lymphocyte activation and induce genes such as *IL2* and *TNF*^11^,^12^. PKC-θ signalling activates a number of transcription factors including the NFκB family members as well as AP1 and NFAT^8^,^13^,^14^, which have a major role in regulating chromatin configuration upon T cell activation^15^,^16^.

Chromatin modifications are different at regulatory regions of memory-responsive genes poised for rapid expression^3^,^17^,^18^. DNA winding around histones affects accessibility to RNA polymerase II (Pol II) and other regulators to control transcription. Histone modifications^19^ and the incorporation of histone variants^20^,^21^ regulate histone-DNA interactions to control chromatin accessibility. Histone modifications such as H3K4me3 and H3K9ac identify “active” transcriptional start sites (TSSs) and gene promoters, while others such as H3K4me1 and H3K27ac identify distal regulatory elements (enhancers)^22^. Formaldehyde-assisted isolation of regulatory elements (FAIRE) is one technique that can measure chromatin accessibility. Combined with high-throughput sequencing (FAIRE-seq), it has been used to demonstrate that chromatin accessibility changes during cellular differentiation in adipocytes^23^, myeloid cells^24^, and breast epithelial cells^25^ largely occur at promoters and enhancers.

To understand the process of T cell response priming, we globally profiled chromatin accessibility by FAIRE-seq in a previously described^7^ human T cell (Jurkat) transcriptional memory (JTM) model of four distinct transcriptional states: 1) non-stimulated cells, 2) stimulated cells, 3) “transcriptionally experienced” or memory-like cells after stimulus withdrawal, and 4) re-stimulated memory-like cells. We found that the initial stimulation alters the chromatin accessibility landscape, especially at regions distant from TSSs. Many of these alterations persisted in the resting memory state, and secondary activation induced secondary-specific opening of previously restricted regions. We identified and characterised regions with enhanced chromatin accessibility that associated with increased expression of memory-responsive genes and identified a role for the combinatory action of transcription factors, unravelling a central role for NFAT and ETS in regulating these regions. Lastly, we perturbed the initial PKC signal and subsequent NFAT and NFκB activation to help dissect the pathways involved in regulating the changes in chromatin accessibility in transcriptional memory. Overall, transcriptional responses are primed via the chromatin accessibility landscape in memory T cells.

## Results

### Primary stimulation irreversibly alters the chromatin accessibility landscape

**Figure 1:**
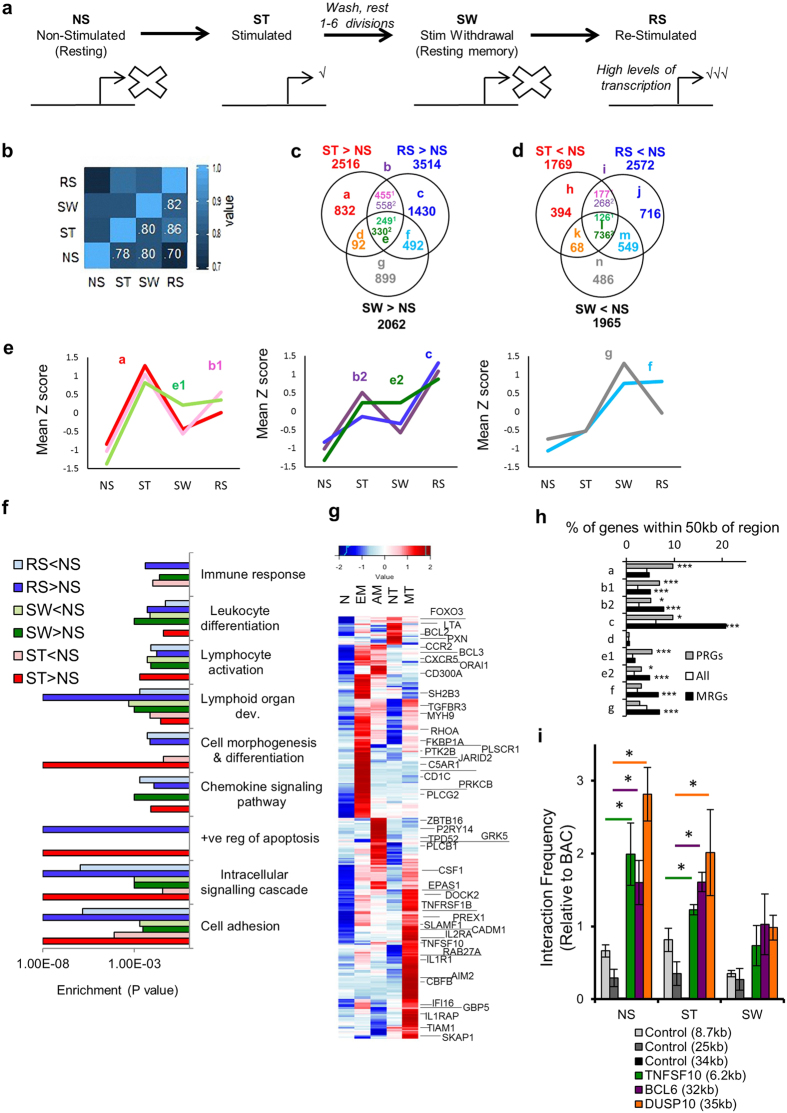
Initial T cell stimulation alters the immune gene DNA accessibility landscape with enduring consequences upon stimulus removal. (**a**) Schematic of the Jurkat transcriptional memory model. Non-stimulated (NS) Jurkat T cells were stimulated with PMA and ionomycin (ST), washed, and rested (SW) before re-stimulation (RS). (**b**) FAIRE-seq Spearman correlation. (**c,d**) Region with more (**c**) or less (**d**) accessibility in ST, SW, and RS cells compared to NS cells. Sets with altered accessibility in RS and ST were divided according to whether the accessibility was higher (≥1.75) in ST (^1^) or RS (^2^). (**e**) Mean Z-score profiles of the increased accessibility sets. (**f**) Over-represented Gene Ontology biological pathways and KEGG pathways in the genes nearest to the regions with increased or decreased accessibility. (**g**) The expression of genes near SW >NS accessible regions that are upregulated in effector memory (EM), activated memory (AM), naïve Tregs (NT), and memory Tregs (MT) compared to naïve (N) (from E-MEXP-2578). Immune response genes are labelled. Heat map Z-score scaled. (**h**) The percentage of primary response genes (PRG), all genes on the array, and memory-responsive genes (MRG) with transcriptional start sites (TSSs) within 50 kb of a region for the different subsets. *p < 0.05, **p < 0.001, ***p < 1 × 10^−5^, compared to all genes on array, Fisher’s exact test.(**i**) Chromatin conformation capture ligation efficiencies indicating interactions between *TNFSF10, DUSP10*, and *BCL6* enhancers and gene TSSs. The contact frequencies of the gene desert region with similar distances were used as a control. 3C-qPCR data were normalised to bacterial artificial chromosome (BAC) clone ligation products (mean ± SEM, n = 4–5 biological replicates, *p < 0.05, *t*-test). Interaction frequency should be considered within a treatment relative to the gene desert region and not across treatments.

We performed genome-wide FAIRE-seq analysis of open chromatin in the Jurkat transcription memory (JTM) model to determine the chromatin accessibility domains marking transcriptional memory-responsive genes. The JTM model^7^ consists of four cell states: NS (non-stimulated naïve T cells), ST (PMA and ionomycin (I) stimulated), SW (stimulus withdrawal, memory-like T cells), and RS cells (re-stimulation of the memory-like T cell state) (Fig. 1a). The cells were pre-treated with DMSO as a vehicle control for the experiments with the PKC inhibitor rottlerin. The stimulation of Jurkat cells globally altered chromatin accessibility (Spearman’s rank correlation 0.78 compared to NS; Fig. 1b), and this overall difference partly persisted in SW (ρ = 0.80). However, the greatest chromatin accessibility changes were detected in RS (ρ = 0.70). For comparison, rottlerin pre-treatment of NS cells, which was not expected to induce changes, had ρ = 0.93 compared to the DMSO pre-treated NS cells.

We next identified regions with increased or decreased chromatin accessibility in ST, SW, and RS cells compared to NS cells (see Methods, Fig. S1A–D) and their overlap (Fig. 1c,d). The 5337 regions with increased chromatin accessibility in ST, SW, and/or RS cells were classified into sets (sets a–g, Fig. 1c,e). Regions in sets b and e showing greater chromatin accessibility in ST and RS cells were further subdivided depending on whether accessibility was greater in ST (b1,e1) or RS (b2,e2). This included patterns with: chromatin accessibility greatest in primary (1°) activation (1° enriched; a, b1,e1); chromatin accessibility greatest in secondary (2°) activation (2° enriched; b2,c,e2); and high chromatin accessibility in SW (f,g). Of the 2516 regions with increased chromatin accessibility in ST (a,b,d,e), 671 retained CA in SW (d,e; Fig. 1c) and 579 regions (e) were common to the ST, SW, and RS samples. A further 1391 regions (f,g) had “delayed” greater chromatin accessibility in SW compared to NS controls, with 492 of these also having chromatin accessibility in RS cells (f, Fig. 1c). In RS, 1013 regions (b) with reversed chromatin accessibility in SW were re-induced, and accessibility increases at 1430 2° specific regions (c) were identified. Regions with decreased chromatin accessibility relative to NS were similarly grouped (h–n) (Fig. 1d). Decreases tended to be less reversible than increases in accessibility. Overall, RS had the greatest number of regions with increased or decreased chromatin accessibility and had more increased regions in common with ST than with SW cells.

Here, FAIRE-seq identified four patterns with enhanced chromatin accessibility in RS: (i) stimulation-dependent chromatin accessibility that is lost in SW but enhanced upon RS (b2); (ii) 2°-specific chromatin accessibility detected only in the RS state (c); (iii) persistent chromatin accessibility that remains in cells from ST and SW but is highest in RS (e2); and (iv) delayed chromatin accessibility that is detected in SW and RS T cells (f).

### Altered chromatin accessibility regions occur near immune pathway genes, with increased chromatin accessibility in RS occurring near memory-responsive genes

We identified the nearest genes to the regions and found that in comparison to regions that exhibited little change in chromatin accessibility, those with differential accessibility were primarily located in intron and intergenic regions (Fig. S1F). Genes near increased chromatin accessibility regions were significantly enriched for TCR and chemokine signalling pathways (Fig. 1f, Fig. S1G). Genes associated with regions that exhibited either increased or decreased accessibility were enriched for immune-related terms including lymphocyte activation, haematopoietic development, regulation of apoptosis, cell morphogenesis, and cell adhesion processes (Fig. 1f). Of note, a subset of immune-responsive genes including *CXCR5, PLCG2, DOCK2, CSF1*, and *BCL3* were associated with increased chromatin accessibility regions in SW cells, and while their transcription did not necessarily change in Jurkat cells, increased transcription was observed in *in vivo* models of T cell memory and/or differentiation (Fig. 1g). This supports a role for the primary TCR signal in changing the plasticity of the chromatin accessibility landscape so that cues such as cytokines can activate signalling pathways whose target transcription factors can then access these opened regulatory regions and activate transcription.

We next used JTM microarray data (GSE61172; same as FAIRE-seq except with 9 day SW) to determine the relationship between chromatin accessibility changes and transcription of memory-responsive genes. As regulatory regions can act on genes up to 750 kb away^25^, we examined the relationship between regions and expression by determining the percentage of memory-responsive genes (expression higher in RS than NS and ST) or “1° response” genes (higher expression in ST than NS and RS) which had TSSs within 50 kb of the region sets (Fig. 1h). Regions exhibiting increased chromatin accessibility in 1° (a,b1,e1) and 2° states (b2,c,e2) demonstrated a greater association with 1° response genes than expected (p < 0.05). There were significantly more memory-responsive genes within 50 kb of all 2° enriched sets, SW enriched sets (f and g), and set b1 than expected (p < 3 × 10^−6^). Genes exhibiting decreased transcription in RS cells (Fig. S1H) were generally associated with regions that exhibited a decrease in chromatin accessibility.

Memory-responsive genes with 2°-specific memory chromatin accessibility regions (c) included *IL8, ID2*, and *FASLG*. Memory-responsive genes with persistent (e2) or delayed memory (f) chromatin accessibility included *MIR21, BIRC3, PRDM8, TNFSF10, DUSP10, MIR155*, and *LTA* (Fig. S2A). The region near *LTA* can enhance *LTA* and *TNF* transcription in reporter plasmids^26^,^27^, and, in stimulated T cells, contacts the *TNF* promoter^27^; we refer to this region as *LTA/TNF*. Interestingly, many memory-responsive genes near these SW accessible regions had low transcription in SW cells (Fig. S2), suggesting that this chromatin accessibility may prime genes for an enhanced response upon RS rather than contribute to transcription in SW.

Contacts between distal regulatory regions and their promoter targets have been reported to strengthen upon stimulation^27^, while others have suggested that contact reflects a preconfigured state for transcriptional poising^28^,^29^,^30^. Chromosome conformation capture (3C) can be used to determine if distal elements are in close contact to the TSSs of particular genes^31^. Using primers targeting three of our memory accessible regions located 6–35 kb from the TSSs of the memory-responsive genes *DUSP10, BCL6*, and *TNFSF10* (Fig. S3), we compared distal TSS interactions of these memory-responsive genes in NS, ST, and SW (6 days) cells. We also used control primers for a gene desert region^32^ to measure background interactions occurring by chance. Interactions were significantly greater in NS and ST cells for *TNFSF10* (p = 0.025 and 0.011) than the 8.7 kb control, and a similar interaction was also detected for *BCL6* (p = 0.021 and 0.002) and *DUSP10* (p = 0.001 and 0.043) compared to the 25 kb control (Fig. 1i). There was no amplification of the 34 kb control region in any treatment. Unexpectedly, interactions were not significant in SW cells, although still stronger on average than control regions. When normalised for control region interactions, changes across treatments were not significant and the differences in control region interactions across treatments indicate that they are incomparable. Thus, at least for the regions examined, the memory accessible enhancer regions interacted with promoters of memory-responsive genes, and these interactions were present in NS cells before increased gene expression upon activation.

### Changes in chromatin accessibility primarily occur in enhancer regions and occur in CD4^+^ memory lymphocytes in selected human individuals

**Figure 2:**
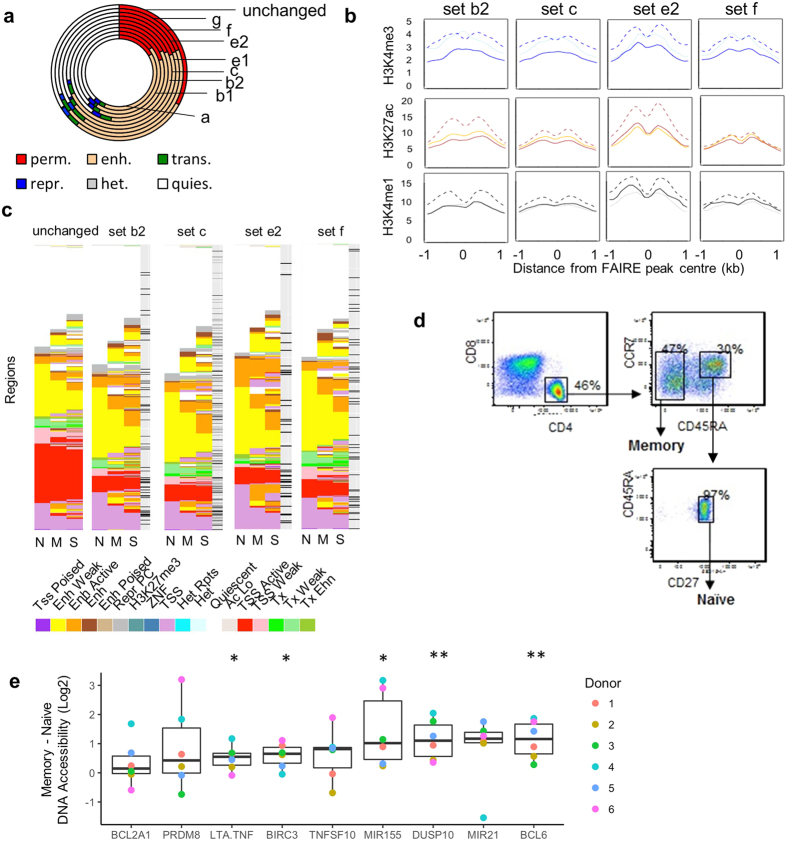
Changes in the chromatin accessibility (CA) landscape occur in regions with enhancer or permissive chromatin environments in primary CD4 lymphocytes. (**a**) Regions in the sets with increased chromatin accessibility were annotated by their chromatin state segmentation in CD4^+^ memory cells. The chromatin state data were summarised from CD4^+^ Roadmap data: quiescent (quies, low level of marks), repressive (repr, H3K27me3), transcription (trans, H3K36me3), enhancer (enh, H3K4me1), permissive (perm, H3K4me3), and heterochromatin (het, H3K9me3). (**b**) H3K4me3, H3K27ac, and H3K4me1levels around the FAIRE peaks in the transcriptional memory sets in naïve (faint line), memory (dark line), and PMA/I stimulated Th (dotted line) lymphocytes. Average profiles are of sequencing tags per 100 bp. (**c**) Detailed chromatin state of the memory regions in naïve (N), memory (M), and PMA/I stimulated Th (S) CD4^+^ lymphocytes. Those regions within 50 kb of an MRG TSS are marked with a black line. Transcribed (Tx), Permissive (Tss), Enhancer (Enh). (**d,e**) Fold change in FAIRE chromatin accessibility of CD4^+^ memory cells compared to naïve cells (Log2). Naïve (CD4^+^ CD45RA^+^ CCR7^+^ CD27^+^) and memory (CD4^+^ CD45RA^−^ blood lymphocytes were isolated from 6 donors (biological replicates). Chromatin accessibility was measured by qPCR and normalised to the *PPIA* promoter. Boxplots show median and first to fourth quartiles (*p < 0.05, **p < 0.01, one sample *t*-test against 0).

As a large proportion of the changes in chromatin accessibility occurred away from a TSS, we examined if they were occurring in genomic regions containing histone marks associated with regulatory elements such as enhancers. We used Roadmap chromatin state annotations^22^ to profile the histone environment of our regions in different primary cell types including CD4^+^ naïve and memory cells. We initially grouped the chromatin states into permissive (H3K4me3), enhancer (H3K4me1/H3K27ac), transcription (H3K36me3), repressive (H3K27me3), heterochromatin (H3K9me3), and quiescent (low or no marks). While 33% of regions with unchanged chromatin accessibility were in permissive regions in CD4^+^ memory cells, less than 21% of any of the sets with increased chromatin accessibility were associated with such permissive regions (Fig. 2a). Instead, larger proportions of sets that exhibited an increase in chromatin accessibility overlapped with enhancer regions (35–50%, Fig. 2a). In contrast to the less cell-specific permissive regions, the majority of enhancer regions with increased chromatin accessibility were more cell specific and only had enhancer chromatin marks in T cells or CD34^+^ progenitor cells (Fig. S4A).

Using the H3K27ac, H3K4me3, and H3K4me1 marks in naïve, memory, and PMA/I-stimulated Th cells from the same donor^22^, we examined the FAIRE peak positions with respect to the modified histones using average ChIP-seq data profiles (Fig. 2b). The modified histones flanked accessible regions, particularly in activated cells. These profile shape changes suggested that chromatin accessibility increases could be associated with flanking nucleosomes becoming more fixed in their positions and the development of a “nucleosome-depleted region” (Fig. 2b). This could at least in part be due to increases in histone marks such as acetylation and/or H3K4 methylation.

Based on relative H3K27ac and H3K4me1 levels, Roadmap divides enhancer states into weak, active and poised. Most accessible regions annotated as enhancers were in a weak state in naïve cells (Fig. 2c). However, in the delayed (f) and persistent memory (e2) sets, 20–22% of the weak enhancers gained H3K27ac in memory cells to become active enhancers. Furthermore, 21–22% of quiescent regions in these sets gained enhancer marks. In contrast, a greater proportion of the weak enhancers in sets with corresponding decreases in chromatin accessibility (m and l2) lost H3K4me1 (18–21%) (Fig. S4B). With respect to the four different memory chromatin accessibility groups (b2,c,e2,f), 34–46% of regions which were weak enhancers in naïve cells became active enhancers in PMA/I-stimulated Th cells, and 17–26% of the quiescent regions had enhancer marks (Fig. 2c). Regions near memory-responsive genes were mainly located in permissive or enhancer environments (Fig. 2c).

To determine if primary memory T cells also showed increased chromatin accessibility at regions with delayed or persistent memory in the JTM, we performed FAIRE-PCR on naïve and memory CD4^+^ cells isolated from six healthy donors (Fig. 2d,e). Nine candidate regions near memory-responsive genes were chosen that had either delayed memory (*TNFSF10, MIR155, DUSP10*), persistent memory (*PRDM8, BCL12A1, LTA/TNF, BIRC3, MIR21*), or otherwise maintained chromatin accessibility in SW cells (*BCL6*). Regions close to *DUSP10, BCL6, MIR155, BIRC3*, and *LTA/TNF* had significantly higher chromatin accessibility levels in memory CD4^+^ cells compared to naïve cells (Fig. 2e). Interestingly, not all donors had increased accessibility for all regions, and different donors did not have increased accessibility detected for different regions, most noticeably donor 4 and *MIR21*. It would be interesting to determine whether this variability reflects differences in memory subsets and if individual memory lymphocytes show variability within the same donor.

Together, the Roadmap analysis and primary cell experiments highlight the overlap between changes in primary cells and the JTM and that changes in chromatin accessibility occur in regions with enhancer or weak permissive chromatin marks.

### Stimulation-induced changes in chromatin accessibility are related to changes in H3 and H2AZ levels and acetylation and persist throughout cell division despite stimulus withdrawal

**Figure 3:**
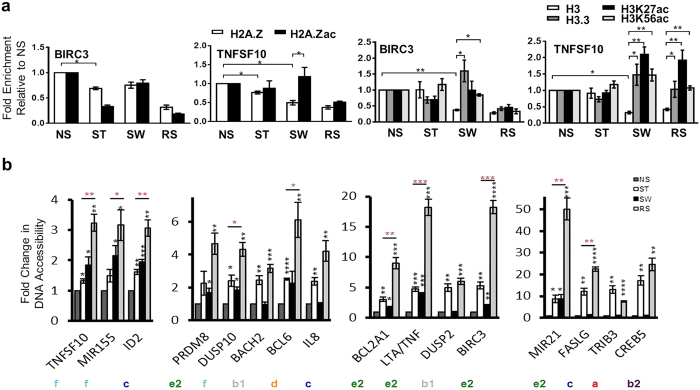
Changes in chromatin accessibility (CA) are associated with altered histone acetylation and variant occupancy and can be remembered through cell division. (**a**) ChIP-qPCR analysis of H2A.Z, H2A. Zac, H3, H3.3, H3K27ac, and H3K56ac binding at the *BIRC3* promoter and *TNFSF10* enhancer in the Jurkat model. ChIP enrichment ratio relative to NS is shown (mean ± SEM, n = 3 biological replicates). *p < 0.05, **p < 0.01, *t*-test. (**b**) FAIRE chromatin accessibility in the Jurkat model with 6 day resting time. Fold change compared to NS. Chromatin accessibility was normalised to the *GAPDH* promoter (mean ± SEM, n = 3 biological replicates, black * compared to NS, red * ST compared to RS, *t*-test, *p < 0.05, **p < 0.01, ***p < 0.001, ****p < 0.0001).

In agreement with other studies^33^,^34^, the chromatin state transitions suggest that histone modifications might mediate a subset of chromatin accessibility changes. Histone variants also influence chromatin accessibility^21^, and we have previously observed variant exchange at inducible gene promoters upon stimulation^35^. We used the promoter of *BIRC3* (e2) and the enhancer of *TNFSF10* (f) as illustrative examples to examine the deposition of H2A. Z and H3.3 variants and the enrichment of tail (H3K27) and globular (H3K56) acetylation (Fig. 3a). H2A. Z but not H3 levels significantly decreased on stimulation (Fig. 3a). In SW, despite significantly lower levels of H2AZ being detected at the *TNFSF10* enhancer region compared to NS, a greater proportion of the H2AZ was acetylated (Fig. 3a). Similar to H2AZ, H3 levels were significantly decreased at both of these regions in SW, but K27 and K56 were highly acetylated and this profile was accompanied by increases in H3.3 deposition (Fig. 3a). There was no increase in the H3K4me1 enhancer mark at the *TNFSF10* region, although there was evidence of relatively higher H3K4me3 in SW cells for the *BIRC3* promoter (Fig. S4C). In RS, H2AZ levels decreased further.

We have previously shown that SW Jurkat cells maintain the ability to induce enhanced *IL2* transcription after several cell divisions^7^. To establish whether increased chromatin accessibility persists through cell division, FAIRE-PCR was performed on SW and RS cells rested for 6 days after stimulation. Jurkat cells stimulated with PMA/I and then washed underwent cell division at least 6 times over the following 6 days (Fig. S2D). In addition to the 9 aforementioned candidate regions, we also examined: 3 with 2°-specific memory near memory-responsive genes (*IL8, FASL, ID2*); 3 (*CREB5, DUSP2*, and *BACH2*) with similar chromatin accessibility in ST and RS that were near inducible genes with similar transcription, and 1 (*TRIB3*) that had higher chromatin accessibility and transcription in ST cells. After 6 days of rest, the response to re-stimulation was significantly higher than that for 1° stimulation for regions from the different memory chromatin accessibility groups except for *PRDM8* and *IL8*, which showed non-significant increases (Fig. 3b). The delayed and persistent memory regions near *TNFSF10, MIR155, DUSP10, BCL2A1, LTA/TNF, BIRC3*, and *MIR21* all maintained significant increases in SW day 6 samples (p < 0.05), with *BCL6* and *PRDM8* showing consistent but non-significant increases (Fig. 3b). In contrast, the 4 control regions and 2 2°-specific regions (*IL8* and *FASLG*) had similar chromatin accessibility levels in the NS and day 6 SW samples (Fig. 3b). Increased chromatin accessibility is, therefore, at least partially maintained throughout at least 6 cell divisions, and the increased chromatin accessibility changes likely reflect decreases in nucleosome occupancy with exchange of H3.3 and relative increases in acetylation of the histones.

### NFAT binding sites are associated with chromatin accessibility in RS cells, and ETS and GATA sites are associated with maintenance of chromatin accessibility

**Figure 4:**
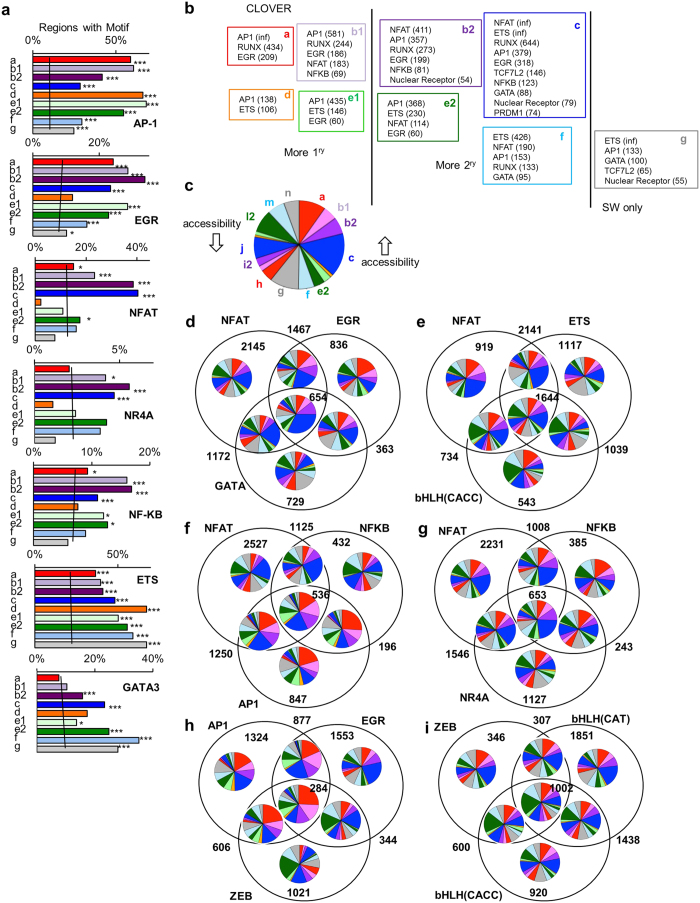
The transcription factor motifs that characterise the different sets of increased chromatin accessibility (CA). (**a**) The top overrepresented motifs in the sets with increased chromatin accessibility. Results from HOMER, *p < 0.05, ***p < 1 × 10^−5^, Benjamini q value, compared to control sets with similar GC content (trend line). (**b**) The top overrepresented JASPAR motif families in the sets. Enrichment scores >50 (scores shown in brackets) and CLOVER significance p value <0.05 relative to NS regions. (**c**) The relative proportions of the region sets with increased or decreased chromatin accessibility. (**d–i**) The numbers of regions with various JASPAR transcription factor binding motif combinations and their chromatin accessibility sets. Regions of increased chromatin accessibility occur first (clockwise).

To identify the transcription factors responsible for changes in chromatin accessibility, we examined the regions for over-represented transcription factor binding motifs (Fig. 4a,b, Fig. S5B). AP1, EGR, RUNX, ETS, NFκB, NFAT, nuclear receptors, TCF7L2 (HMG), and GATA DNA-binding motif families were significantly enriched in regions with increased chromatin accessibility, but their enrichment altered over the region sets (Fig. 4a,b). AP1, EGR, NFκB, and NFAT were most enriched in sets with increases in ST and/or RS cells, with AP1 enriched more in the 1° sets (a,b1,d, and e1) with higher chromatin accessibility in ST cells and NFAT more in sets b2 and c with higher chromatin accessibility in RS cells (Fig. 4a,b). Examination of expression of the factors that bind these motifs indicated that many were inducible (Fig. S5A). Differential induction of several of the factors that bind the AP1 motif, including *ATF3* and *FOS*, in ST and RS cells may contribute to differences in the chromatin accessibility patterns of the various AP1-containing regions. Interestingly, the ETS motif was more enriched in sets c-g, particularly those that maintain chromatin accessibility in SW cells (Fig. 4). Similarly, the GATA motif was most enriched in sets e2, f, and g.

By comparison, bHLH family motifs were enriched in decreased chromatin accessibility regions (Fig. S5B,C). Unlike other families, there were differences between different members. While at least 3 of the factors that bound to this motif family - *TCF3, TCF12*, and *TFAP4 -* decreased expression with stimulation, the altered chromatin accessibility may also have been due to increased expression of the inhibitory ID family, which also bind the motif (Fig. S5A).

Many motif combinations increased the relative percentage of regions in a given set. Set c comprised 16% of all regions in sets a-n (Fig. 4c), a percentage that increased in regions containing both NFAT and GATA or EGR and GATA (Fig. S5) and even further in regions containing all 3 motifs (Fig. 4d). The proportion of set c regions also increased in those containing NFAT and ETS but not bHLH (CACC) (Fig. 4e). Strikingly, when we examined every combination of regions that contained one of the motifs of interest but lacked the NFAT motif, there was a marked decrease in the percentage of regions accessible more in RS (b2, c) (Fig. S6). The co-occurrence of NFAT and NFκB not only favoured increased chromatin accessibility in 2° regions (Fig. 4f,g), but also had the highest proportion of regions near a memory-responsive gene, and 11% of regions with NFAT, NFκB, and NR4A were near a memory-responsive gene.

For AP1-containing regions, co-occurrence with NFAT and/or NFκB increased the proportion of regions in b2 (Fig. 4f), while co-occurrence with ZEB and/or EGR increased set b1 and a proportions (Fig. 4h). Co-occurrence of ZEB appeared to influence bHLH-containing regions, resulting in more set l2 (Fig. 4i).

In summary, different transcription factor family combinations decorate regions accessible in ST, SW and RS. AP1 and bHLH have dominant roles in respectively regulating increased and decreased chromatin accessibility upon ST, while ETS and GATA are associated with chromatin accessibility in SW and NFAT is strongly linked to enhanced chromatin accessibility upon RS.

### The enhanced memory response of several genes is ETS-1 dependent

**Figure 5:**
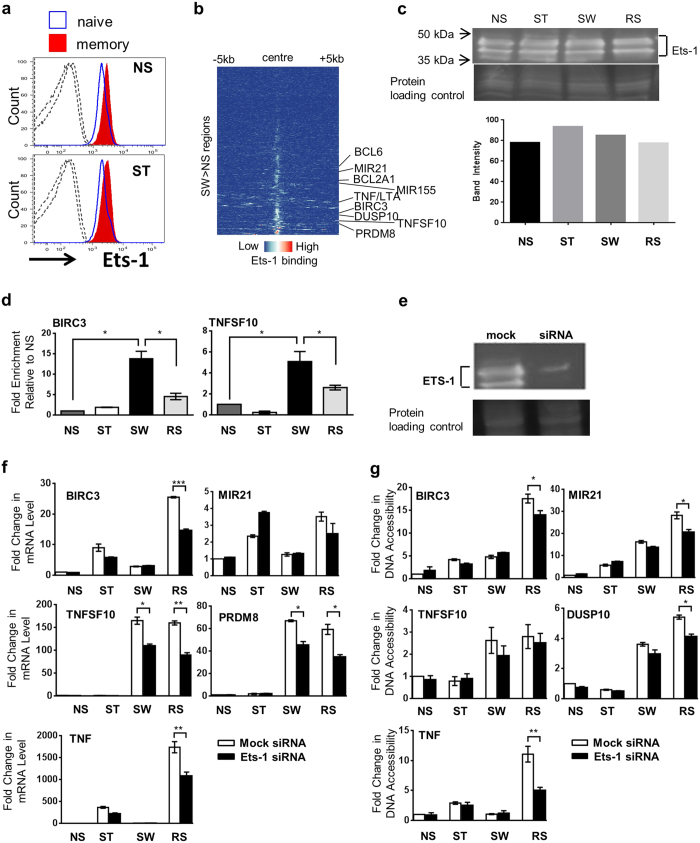
The role of ETS-1 in transcriptional memory. (**a**) Flow cytometric analysis of ETS-1 expression in peripheral blood lymphocytes, naïve (blue, CD45RO^−^), and memory (filled red, CD45RO^+^) CD4^+^ T cells. Cells were left non-stimulated (NS) or stimulated with PMA and calcium ionophore (ST). Dashed lines show isotype control staining. Cells were CD3^+^CD8^−^. A representative of 4 individuals (biological replicates) is shown. (**b**) ETS-1 binding in the memory regulatory regions (SW >NS, sets d-g) in CD4^+^ T cells (GSE43119). Sequencing tags were binned by 100 bp and shown ± 5 kb from the centre of the FAIRE region. (**c**) Immunoblot of ETS-1 protein levels in nuclear extracts of NS, ST, SW, and RS cells. ETS-1 intensities were normalised against Novex (protein loading control). A representative of 3 independent biological experiments is shown. (**d**) ETS-1 binding at the *BIRC3* promoter and *TNFSF10* enhancer in the Jurkat model. ChIP enrichment ratio relative to NS. SW cells were rested for 6 days. A representative (mean+/−SEM with n = 3 PCR (technical) repeats) of 3 independent biological experiments is shown. (**e**) ETS-1 protein levels and Novex (protein loading control) in Jurkat cells with mock or *ETS1* siRNA. (**f**) Expression of putative ETS targets in mock or ETS-1 siRNA-treated Jurkat cells. SW cells were rested for 3 days. mRNA levels were measured by RT-PCR and normalised to *GAPDH*. Fold change relative to mock NS is shown. A representative (mean+/−SEM with n = 3 PCR (technical) repeats) of 5 independent biological experiments is shown. (**g**) Chromatin accessibility (CA) of the *BIRC3, MIR21*, and *TNF* promoters and *TNFSF10* and *DUSP10* enhancers in mock or ETS-1 siRNA-treated Jurkat T cells. SW cells were rested for 3 days. Chromatin accessibility was measured by FAIRE-qPCR and normalised to the *GAPDH* promoter. Fold change relative to mock NS. A representative (mean+/−SEM with n = 3 PCR (technical) repeats) of 3 independent biological experiments is shown. (**d,f,g**) *p < 0.05, *t*-test.

As motif analysis suggested a role for ETS family members in maintaining chromatin accessibility (Fig. 4) and *ETS1* was highly expressed (Fig. S5A), we examined the role of ETS-1 in chromatin accessibility and gene expression. ETS-1 protein was expressed in NS and ST human primary CD4^+^ naïve and memory cells (Fig. 5a), and ETS-1 ChIP-seq of CD4^+^ cells (GSE43119^36^) showed that ETS-1 binds to about half of SW >NS regions (Fig. 5b). ETS-1 protein was present in NS cells and remained stable in ST (Fig. 5c). However, ETS-1 binding at the *BIRC3* promoter and *TNFSF10* enhancer region was highest in SW (Fig. 5d).

To assess the contribution of ETS-1 to the memory response, *ETS1* was knocked down (Fig. 5e). The enhanced expression of *BIRC3, MIR21, TNFSF10, TNF,* and *PRDM8* in RS cells was consistently reduced in the presence of *ETS1* siRNA (Fig. 5f), with decreased expression of *TNFSF10* and *PRDM8* in SW cells. The effects of *ETS1* knockdown on chromatin accessibility were subtler, but consistent chromatin accessibility decreases were observed on RS at the *BIRC3, TNF* and *MIR21* promoter regions and the *DUSP10* enhancer (Fig. 5g). Thus, ETS-1 may bind at regions accessible in SW and play a role in enhancing the recruitment of stimulation-induced factors to these regions upon RS.

### Abrogation of initiating stimuli by rottlerin inhibits aspects of the enhanced 2° response

**Figure 6:**
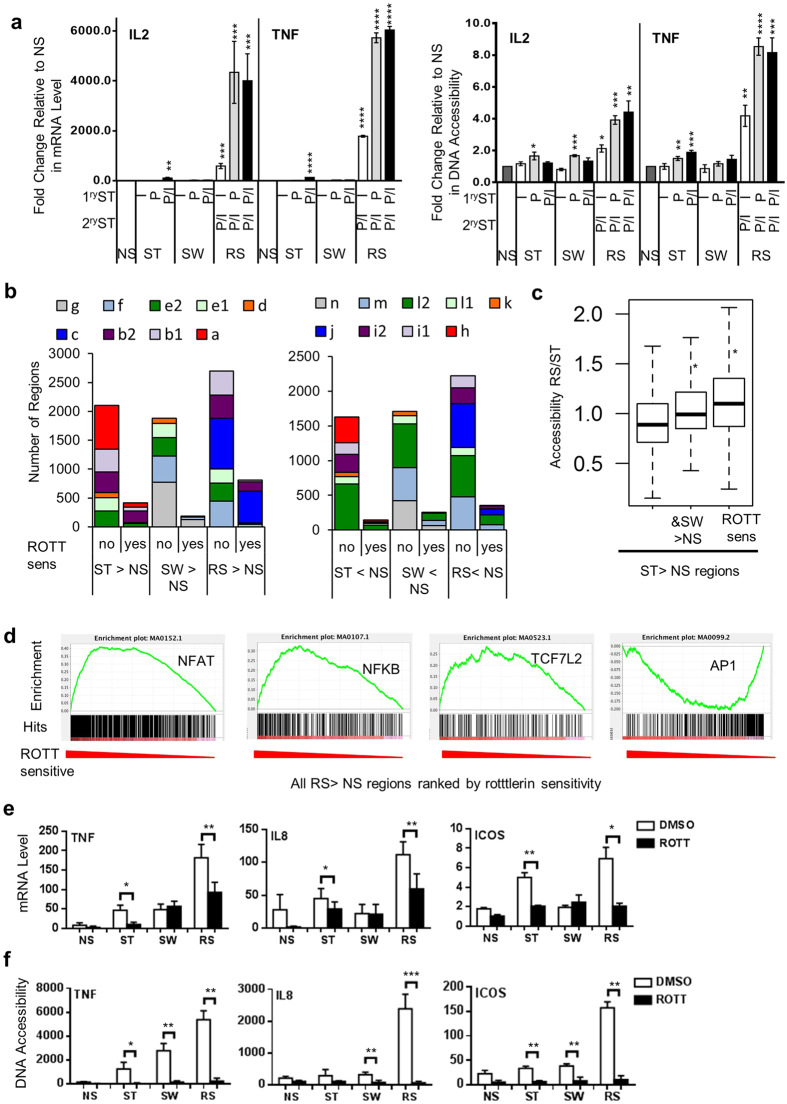
Altering the initial activation signal selectively affects transcriptional memory. (**a**) Transcription of *IL2* and *TNF* and chromatin accessibility (CA) of their promoters in the Jurkat TM model with different initial stimuli. Expression levels were measured by RT-PCR and normalised to *GAPDH*. Chromatin accessibility was measured by qPCR and normalised to the *GAPDH* promoter. *IL2* expression values are mean ± SEM, n = 3 biological replicates. For *TNF* expression, values are the average of the RT-PCR (technical) replicates, and error bars indicate min-max. Accessibility: mean ± SEM, n = 3 biological replicates. *p < 0.05 compared to NS, *t*-test. (**b**) The regions with altered chromatin accessibility in ST, SW, or RS compared to NS and whether their increase or decrease was rottlerin sensitive (ROTT sens). The regions are coloured by their chromatin accessibility set. (**c**) The chromatin accessibility ratio in RS/ST of all regions with increased accessibility in ST >NS compared to those also SW >NS and those rottlerin sensitive in ST. *p < 0.05 compared to all ST >NS, Wilcoxon test. Chromatin accessibility from FAIRE-seq. (**d**) Regions with increased Chromatin accessibility (RS >NS) were analysed for the occurrence of DNA motifs. Regions were ranked according to the ratio of their chromatin accessibility in rottlerin-treated RS cells (compared to DMSO RS), the occurrence of the selected motif is illustrated by black lines and the enrichment score is plotted against the rank of the regions. (**e–f**) The effect of rottlerin (ROTT) pre-treatment on mRNA expression (**e**) and chromatin accessibility (**f**) of the regulatory regions near the gene. Expression levels were measured by RT-PCR and normalised to *GAPDH*. Chromatin accessibility was measured by qPCR and normalised to the *GAPDH* promoter. *p < 0.05, **p < 0.01, *t*-test, n = 3 biological repeats, mean ± SEM.

PMA activates several PKC isoforms including PKC-θ, which plays a central role in T cell activation^8^. Treatment with PMA/I induces transcription of genes such as *IL2* and *TNF* associated with T cell activation^11^,^12^. *IL2* and *TNF* have higher persistent expression in stimulated memory than naïve CD4^+^ cells^2^. We used FAIRE-PCR to examine the role of PMA- and I-driven signals in increasing chromatin accessibility at the *IL2* and *TNF* promoters in ST and RS cells (Fig. 6a, Fig. S7A).

*IL2* and *TNF* expression was higher in RS cells (Fig. 6a), and chromatin accessibility induced in RS was greater than in ST (Fig. 6a). PMA alone increased chromatin accessibility upon initial stimulation (Fig. 6a) and was sufficient to prime cells for enhanced expression in RS even in the absence of increased initial expression (Fig. 6a).

To further dissect the roles of different T cell signalling pathways in regulating chromatin accessibility, FAIRE-seq was performed on cells pre-treated with the PKC inhibitor rottlerin before 1° stimulation and washed away before 2° stimulation (Fig. S7B). Rottlerin inhibited both an increase and decrease in chromatin accessibility at selected genomic regions in ST, SW, and RS (Fig. S7C,D). In particular, rottlerin treatment influenced a greater proportion of chromatin accessibility increases in RS than ST, with the majority of rottlerin-sensitive regions being from two transcriptional memory sets: 2°-specific set c and stimulation-dependent set b2 (Fig. 6b). Furthermore, those regions with rottlerin-sensitive chromatin accessibility increases in ST had significantly higher chromatin accessibility in RS than ST compared to all ST >NS regions (p < 2 × 10^−16^, Fig. 6c), suggesting that the rottlerin sensitive pathways were the same as those enhanced upon RS.

We next used an enrichment approach to identify which transcription factor pathways were affected by rottlerin. When the RS >NS regions were ranked by their chromatin accessibility with and without rottlerin, the NFAT, NFκB, and TCF7L2 motifs were significantly enriched in the regions with higher chromatin accessibility in the absence of rottlerin (p ≤ 0.002, Fig. 6d, Table S1). Interestingly we found that chromatin accessibility at AP1-containing regions was less rottlerin sensitive (Fig. 6d).

Several memory-responsive genes were within 50 kb of a rottlerin-sensitive RS >NS region (Fig. S8). The enhanced 2° expression of three of these memory-responsive genes - *TNF, IL8*, and *ICOS* - was also rottlerin sensitive (Fig. 6e), and we confirmed the rottlerin sensitivity of the chromatin accessibility of the regions using qPCR (Fig. 6f). All three regions contained an NFAT motif (Fig. S8).

**Figure 7:**
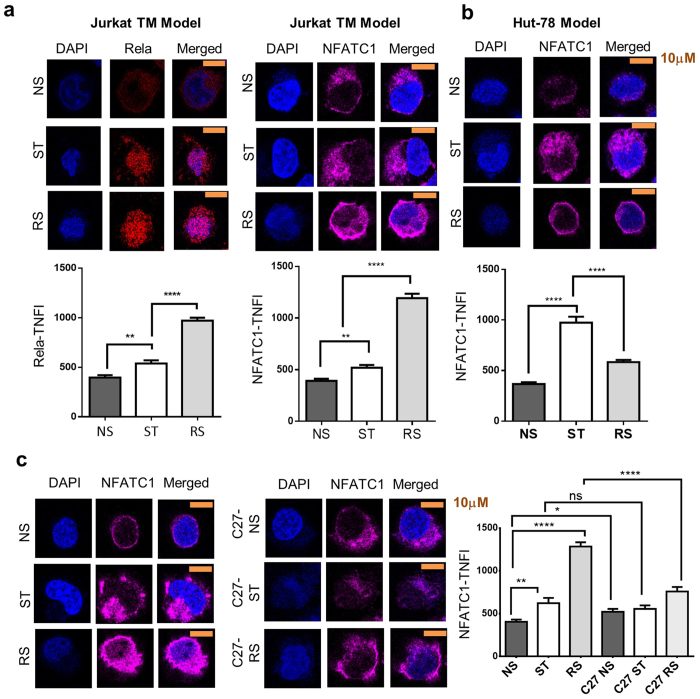
Cellular localisation and levels of NFATC1 in transcriptional memory and its dependence on PKC. (**a**) The nuclear levels of RelA and NFATC1 in P/I stimulated (ST) and re-stimulated (RS) Jurkat cells. (**b**) The nuclear levels of NFATC1 in P/I stimulated (ST) and re-stimulated (RS) Hut-78 cells. (**c**) The nuclear levels of NFATC1 in Jurkat cells pre-treated with DMSO or the PKC-θ-specific kinase inhibitor C27. (**a–c**) Cells were rested for 1 day after the initial stimulation. DAPI co-staining was used. Representative cells for each treatment (10 μm scale bar) are shown, with total nuclear fluorescence intensity (TNFI) from 3 biological replicates with n = 20 cells each, mean ± SEM. *p < 0.05, **p < 0.01, ****p < 0.0001. Mann–Whitney test.

We have previously reported^7^ that nuclear NFκB family member RelA protein levels are higher in 2°-stimulated Jurkat cells, and this was confirmed by immunohistochemistry (Fig. 7a). Furthermore, nuclear NFATC1 levels were also significantly higher in RS cells (Fig. 7a). Nuclear NFATC1 did not increase in RS Hut-78 cells (Fig. 7b), which lack transcriptional memory (unpublished observation). We found that the PKC inhibitor C27^37^ abrogated the enhanced protein levels of NFAT in the nucleus on RS (Fig. 7c).

Taken together, perturbation of the 1° signal in our model using the inhibitor rottlerin revealed two interesting findings. Firstly, that there is overlap in the signalling affected by rotterlin, primarily NFAT and NFκB activation, and the signalling enhanced upon RS. Secondly, that the signalling occurring in the initial activation had consequences for the subsequent enhancement of chromatin accessibility and transcription increases upon 2° stimulation.

### The enhanced 2° response of NFAT is ETS dependent

**Figure 8:**
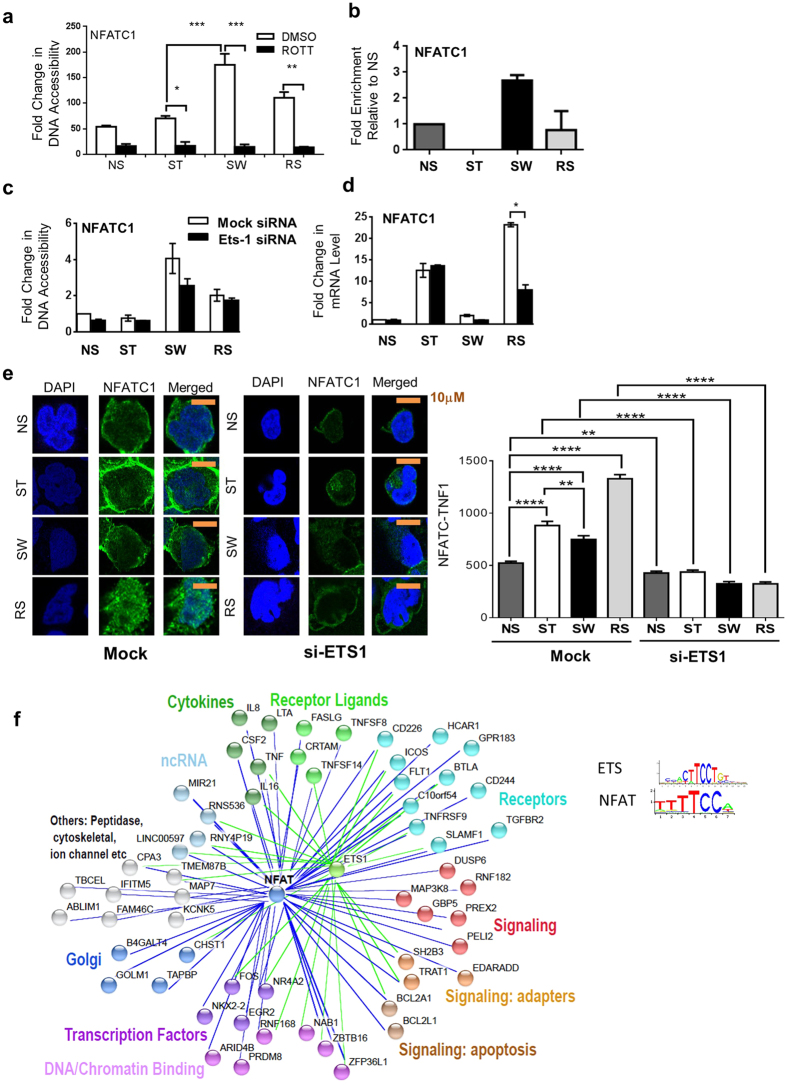
The role of PKC and ETS-1 in regulating NFATC1. (**a**) The effect of rottlerin (ROTT) in the Jurkat transcriptional memory model on DNA accessibility at an *NFATC1* enhancer region. Mean+/− SEM with n = 3 biological repeats. * *p* < 0.05, **p < 0.01, ***p < 0.001. *t*-test. (**b**) ETS-1 binding at the *NFATC1* enhancer region in the Jurkat memory model. Error bars show min-max of 2 PCR values, with the result representative of 3 biological repeats. (**c**,**d**) The effect of *ETS1* siRNA knockdown in the Jurkat transcriptional memory model on accessibility at an *NFATC1* enhancer region (**c**) and *NFATC1* mRNA expression (**d**). Representatives (mean+/−SEM with n = 3 PCR (technical) repeats) of 3 independent biological experiments are shown. (**e**) The nuclear levels of NFATC1 in P/I stimulated (ST), SW, and re-stimulated (RS) Jurkat cells pre-treated with mock or ETS1 siRNA. SW cells were rested for 3 days. Representative cells are shown (10 μm scale bar), with total nuclear fluorescence intensity (TNFI) from 3 biological replicates with n = 20 cells each mean ± SEM. *p < 0.05, **p < 0.01, ****p < 0.0001. Mann–Whitney test. (**f**) The memory responsive genes within 50 kb of a region with rottlerin sensitive increased accessibility in RS and that contained a NFAT motif. Regions that also contained ETS-1 motifs are indicated with green lines.

Microarray data suggested that enhanced nuclear NFATC1 protein expression in RS cells was due to increased *NFATC1* gene expression (Fig. S4A). Chromatin accessibility at the *NFATC1* promoter was not maintained in SW cells, but several small chromatin accessibility peaks were present in SW FAIRE-seq upstream of the TSS, with at least one in an area with enhancer chromatin marks in primary CD4^+^ lymphocytes (Fig. S9A). We confirmed increased chromatin accessibility in SW cells of a region with enhancer chromatin marks by qPCR and found that rottlerin pre-treatment significantly abrogated chromatin accessibility (Fig. 8a). Given that ETS-1 was strongly associated with chromatin accessibility in SW cells, we investigated its role in maintaining chromatin accessibility at this enhancer region. As hypothesised, ETS-1 bound to this region (Fig. 8b), and *ETS1* knockdown abrogated the chromatin accessibility on serum withdrawal (Fig. 8c). Future experiments would allow confirmation its interaction with the *NFATC1* promoter. ETS1 knockdown also partially inhibited the enhanced *NFATC1* mRNA (Fig. 8d) and nuclear protein levels (Fig. 8e) on RS. Interestingly, ETS-1 knockdown decreased the level of NFATC1 protein in the cytoplasm and nucleus (Fig. S9B, Fig. 8e) for all treatments, while its effect on mRNA was limited to RS (Fig. 8d).

In addition to regulating *NFATC1* expression, the ETS binding motif co-existed with the NFATC1 motif in many increased chromatin accessibility regions in RS cells (Fig. 4e). Memory-responsive genes with rottlerin -sensitive RS regions and NFAT motifs play important roles in the immune response and include transcription factors, cytokines, receptors, and signalling molecules (Fig. 8f). Interestingly, many of these regions also contained an ETS motif, with many containing an overlapping NFAT-ETS motif (Fig. 8f). Thus, ETS together with PKC pathway-induced factors may promote NFAT activity in several different ways that together play a major role in the enhanced chromatin accessibility and transcriptional 2° response of pre-activated T cells.

## Discussion

Various immune cells maintain memory of previous activation and produce different transcriptional responses upon 2° activation than occur upon 1° activation^34^,^38^. There is growing evidence that this transcriptional memory is encoded in the epigenome^18^,^34^,^38^,^39^. Here we used a human T cell culture model with stimuli acting downstream of cell surface signalling to explore if the chromatin accessibility landscape “remembers” previous activation and to identify the transcription factors required to establish and enhance the chromatin accessibility changes associated with rapid gene expression upon re-stimulation. In doing so, we highlight the likely combinatory action of transcription factors in regulating inducible genes with memory.

We found that the transcription of many memory-responsive genes corresponded to regions with increased chromatin accessibility in RS, which were often located away from the transcriptional start site in cell type-specific enhancer regions. Studies using a *TNF* enhancer region have shown it to interact more strongly with the *TNF* promoter in PMA/I-stimulated T cells^27^. However, examination of three interactions in our model revealed that these enhancer regions were already in close proximity to transcriptional start sites in naïve T cells. Several other studies have found evidence for promoter-enhancer interactions in the absence of gene expression^30^,^40^,^41^, suggesting the existence of pre-established (“permissive”) and *de novo* (“instructive”) loops^42^. Most similar to our study, inducible genes such as *CCL2* already had enhancer-promoter loops in the absence of the TNF treatment that induces their expression. The three interactions we examined appear to be “permissive loops”, with increased accessibility at the enhancers in the SW state a secondary step and RS-dependent factors a tertiary step to enhanced expression. Although beyond the scope of the current study, It would be informative to examine additional enhancers and to utilise other methods such as ChiA-PET to investigate interactions in the JTM.

In examining all the different chromatin accessibility change profiles in our JTM model, we identified four groups with greater chromatin accessibility in RS than ST: (i) stimulation-dependent (set b2); (ii) persistent transcriptional memory (set e2); (iii) delayed and maintained response (set f); and (iv) 2°-specific (set c). We propose that all four groups contribute to memory-responsive gene induction and, in agreement with others^38^, that different regions act together to modulate transcription. For instance, *NFATC1, FASLG, TNFSF10*, and *DUSP10* had multiple nearby chromatin accessibility regions. It is increasingly apparent that not only do multiple regions regulate transcription of a target gene, but that chromatin accessibility at each of these regions is regulated by a combination of transcription factors that contribute differently at various activation stages.

**Figure 9:**
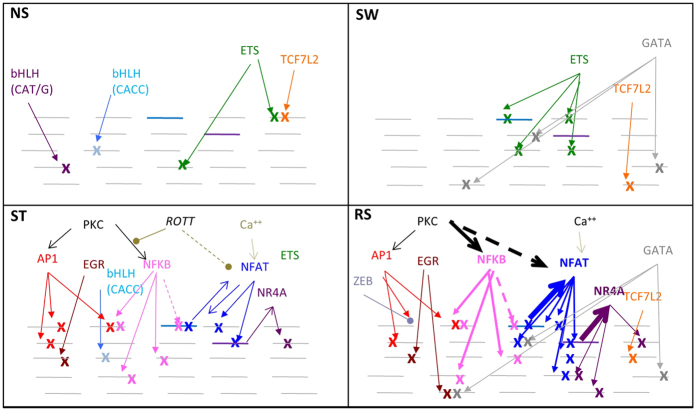
Schematic of how transcription factors act in combination to regulate chromatin accessibility (CA) in transcriptional memory. In non-stimulated (NS) cells, members of the bHLH, ETS, and TCF7L2 families maintain chromatin accessibility regions. On stimulation (ST), PKC and calcium signalling pathways activate the AP1, EGR, NFκB, NFAT, and NR4A families, which increase accessibility and de-activate some signalling by bHLH members. When stimulus is withdrawn (SW), activation of the induced transcription factors ceases but GATA family members gradually build up and, with ETS family members, maintain a subset of the chromatin accessibility regions and make additional regions accessible. These regions include a potential *NFATC1* enhancer. On re-stimulation (RS), there is re-activation of the induced transcription factors with increased activation of NFκB, NFAT, and NR4A, leading to increased chromatin accessibility at some regions that had increases before and secondary-specific chromatin accessibility at additional regions. The factor ZEB is also activated more upon RS and inhibits accessibility, while differential expression of ID proteins and bHLH members lead to decreases in chromatin accessibility in SW and RS. Weakening of the initial PKC signal by rottlerin (ROTT) partially inhibits chromatin accessibility induced by NFκB and NFAT in the primary and subsequent stimulation. Increased chromatin accessibility marked by x. Purple line represents *NR4A* genes, blue line is *NFATC1* gene.

The stimulation-dependent (set b2) group contained motifs for transcription factor families such as NFAT, NFκB, NR4A, and AP1. For this set, the most likely cause of increased chromatin accessibility in RS cells is increased activation of these transcription factors (Fig. 9). While chromatin accessibility at these regions increased in RS compared to ST cells, the increases were not maintained in SW cells when nuclear levels of these transcription factors decreased. It is possible that, as in macrophages, H3K4me1 is maintained at these regions^34^ and some of these regions change chromatin state in memory CD4^+^ lymphocytes.

The persistent (set e2) and delayed (set f) group were enriched for the ETS motif. ETS-1 is constitutively expressed in Jurkat cells, and our results suggest it may play a role in chromatin accessibility in NS in regulatory regions that lose chromatin accessibility upon stimulation (such as set h). Similar to the role of the ETS family member PU.1 in macrophages^34^, some regions appeared to require other transcription factors to modify the chromatin at regions before ETS-1 can bind. Our work and that of ref. 38 suggest that, in lymphocyte activation, members of the AP1, NFAT, EGR, and NFκB families execute this role. AP1 has previously been shown to tether ETS-1 to genes such as *TIMP1*^43^. However, once ETS-1 binds DNA, it appears to be able to maintain chromatin’s open state.

Consistent with its role in memory lymphocyte enhancer regions^36^ and in agreement with the murine lymphocyte study of ref. 38, ETS-1 binding was increased in pre-activated resting lymphocytes, suggesting a role for ETS-1 in maintaining chromatin accessibility (Fig. 9). A recent study^44^ on chromatin accessibility in Tregs showed that ETS bound sites were accessible before Foxp3 was present and that ETS might keep these sites open for Foxp3. At the *NFATC1* enhancer, *ETS1* knockdown partially inhibited chromatin accessibility at regions such as the *BIRC3* promoter, but, surprisingly, had more of an effect on increasing chromatin accessibility in RS than SW cells. This could be due to incomplete knockdown or redundancy with other ETS family members such as ELF-1 or ETV5, or it could suggest a more complex role for ETS in regulating increased chromatin accessibility in RS. ETS-1 and other ETS family members are known to recruit histone acetylases^45^,^46^, and the presence of ETS-1 may affect histone acetylation in SW cells and play a role in recruiting factors that control enhanced chromatin accessibility in RS or recruiting other transcription factors.

ETS motifs also marked the 2°-specific set (c). However, the NFAT motif appeared to be most critical for determining these regions, and several known NFAT targets including *CSF2, EGR2, NR4A2, IL8, FASLG*, and *TNF* are near these regions^15^,^47^,^48^,^49^. Alone, NFAT members only weakly bind DNA, and its regulation of transcription co-depends on AP1, GATA, and EGR members^48^,^50^, a phenomenon highlighted by our examination of motif co-occurrence in set c with NFκB, NR4A, GATA, and EGR motifs also being enriched. Different transcription factor combinations may also continue to affect the immunological outcome, such that NFAT-AP1 co-binding induces immune response genes, while NFAT binding without AP1 induces anergy-associated genes^47^.

The activities of several members of the NFAT family (except for NFAT5) are regulated by calcium signalling^48^. *NFAT5* and *NFATC1* show inducible expression^51^, with *NFATC1* induced more in CD4^+^ memory cells than naïve cells^7^,^38^,^52^. *NFATC1* and *NFAT5* were both induced in Jurkats, with *NFATC1* induced more in RS. *NFATC1* induction is under the control of two alternative promoters and at least one intronic enhancer^53^. While auto-regulation plays a large role in the induction of *NFATC1*, NFκB has also been shown to bind to and regulate one of the promoters (P1)^51^,^53^. Increased NFAT activation upon RS may be partly due to higher NFκB activity acting through P1. There was more nuclear RelA protein accumulation and higher expression of *REL* and *NFKB1* in RS in the JTM, consistent with greater NFκB activity on activation in primary memory than naïve cells^54^. However, it is likely that there is more than one cause of enhanced NFATC1 induction in memory T cells. We also identified a region upstream o*f NFATC1* that became accessible in SW cells and has enhancer chromatin marks in CD4 lymphocytes; we propose that this could act as an *NFATC1* enhancer, although further experiments would allow confirmation of its interaction with the *NFATC1* promoter. This region was bound by ETS-1 in SW cells and may be responsible for the ETS-1-dependent *NFATC1* induction. While the decreased nuclear levels of NFATC1 in RS cells on ETS1 knockdown could be attributable to decreased transcript, the inhibitory effect in ST cells appeared to be independent of mRNA levels. This may implicate ETS-1 and/or its targets in the control of NFAT protein stability.

In addition to regulating *NFATC1* mRNA, ETS-1 and NFAT physically interact in T cell nuclei, and NFAT recruitment to the *IL2* promoter decreases in the absence of ETS^55^. There is partial ETS and NFAT binding motif overlap (Fig. 8f), resulting in many NFAT targets containing composite sites. Hence, increased ETS-1 in SW may help to recruit NFAT more quickly to the enhancers of memory-responsive genes.

NFAT is implicated in regulating chromatin accessibility via recruitment of remodellers BRG and BRM^56^ and histone modifiers such as p300^57^. Acetylases such as p300 are thought to maintain transcriptional memory^39^. We found that increased chromatin accessibility was associated with the development/widening of an NFR, increased relative H3.3 levels, and, in some cases, relative histone acetylation. Interestingly, these changes were inherited through cell divisions. This is unlikely to be due to DNA methylation due to the low CG content of the regions (results not shown) but is thought to be linked to BRD4 binding^38^.

Our motif co-occurrence analysis showed that an important determinant of enhanced chromatin accessibility upon re-stimulation was the absence of a bHLH or ZEB motif. The repressors *ZEB2* and *ID2* are memory-responsive genes in the JTM model and *ZEB2* is an NFAT target^47^. There was further decreased chromatin accessibility of regions with the ZEB binding motif in RS or increased chromatin accessibility only in ST (Fig. 9). Thus, NFAT may also indirectly regulate the further decrease in chromatin accessibility in RS.

In addition to being dependent on ETS-1, *NFATC1* enhancer chromatin accessibility was dependent on PKC signalling in ST. Partial abrogation of the initial PKC signal by rottlerin not only affected chromatin accessibility in ST but continued to affect chromatin accessibility in SW and RS. The regions most sensitive to rottlerin in RS were marked by NFAT and NFκB motifs, while fewer of those with AP1 motifs were affected. We found that NFAT nuclear accumulation was dependent on PKC-θ signalling using the C27 inhibitor. NFκB inhibition by rottlerin was expected, as NFκB is a known PKC signalling target^13^. We previously^7^ found that stimulation-dependent *NFATC1* expression in CD4^+^ naïve and memory T cells is PKC-θ dependent by siRNA knockdown. Furthermore, the PKC-θ inhibitor AEB071 inhibits NFκB and NFAT but not AP1 activity^58^. The results with rottlerin suggest that naïve cells exposed to different initial stimuli may be primed differently for gene expression upon re-stimulation. Differential TCR signalling strength and quality affect not only the development of the memory phenotype, but also T_CM_/T_EM_ balance^14^,^59^. It would be informative to study the epigenomes of lymphocytes with differing activation signals to determine if they are poised differently. Indeed, it was interesting that we observed differences in the relative accessibility in memory and naïve lymphocytes between individuals. The extent of these differences and how they relate to the individual’s ability to induce the corresponding genes remains to be determined. Differences in the CD4+ lymphocyte epigenomes of individuals have been found to relate to factors such as disease status and age^60^,^61^,^62^ and factors such as T_CM_/T_EM_ balance may also play a role.

In conclusion, we show that T cell activation irreversibly changes the chromatin landscape and affects subsequent gene expression profiles if the cells are re-stimulated. NFATC1 and ETS play important roles in enhanced 2° responses, and the strength of initial PKC signalling affects chromatin accessibility changes via NFAT and NFKB motifs.

## Methods

### Jurkat T cell culture

Human Jurkat T cells (Clone E6-1, ATCC TIB-152) were pre-treated with either DMSO, 15 μM rottlerin (Calbiochem, Merck Millipore, Billerica, MA), or 1 μM C27 (SYNthesis med chem, Parkville, Victoria) for 1 h (rottlerin) or 2 h (C27) followed by stimulation with 24 ng/mL phorbol 12-myristate 13-acetate (PMA; Sigma-Aldrich, St. Louis, MI) and/or 1 μM calcium ionophore (I; Sigma-Aldrich) for 2h at a density of 5 × 10^5^ /mL. Primary activated Jurkat T cells were washed three times with stimulus-free medium and re-cultured for up to six days prior to PMA/I re-stimulation as previously described^7^. Jurkat and HUT-78 cells were cultured in RPMI 1640 supplemented with 10% FBS and 4 mM L-glutamine.

### Human T cell isolation

Human blood was obtained from healthy volunteers recruited after obtaining informed written consent in accordance with QIMR Berghofer Medical Research Institute Human Research Ethics Guidelines and approved by the QIMR ethics committee, ethics approval number P158. Peripheral blood mononuclear cells (PBMCs) were isolated from heparinised blood by Ficoll-Paque PLUS (GE Healthcare, Little Chalfont, UK) density centrifugation and labelled with CD8α-PerCP, CD4-PE Cy7, CD3-APC, CCR7-Alexa Fluor700, and CD45RA-FITC (BD Biosciences, San Jose, CA) antibodies and subsequently sorted for CD4^+^ naïve (CD45RA^+^ CCR7^+^ CD27^+^) and memory (CD45RA^−^) cells using the FACS Aria (BD Biosciences) cell sorter.

### Flow cytometry

For carboxyfluorescein succinimidyl ester (CFSE) analysis, previously stimulated (2 h) Jurkat cells were incubated with CFSE (10 μM) in PBS (with FCS 5%) for 10 min before being washed and re-cultured in RPMI. Fluorescence was measured using the LSRII flow cytometer (BD Biosciences) and analysed with FlowJo. The background fluorescence of unstained cells was subtracted from each intensity measurement, and the values were compared to day 0 cells. One cell division was presumed to be equal to a 2-fold intensity decrease.

For Ets-1 intracellular staining, PBMCs from healthy volunteers were stained with CD3 AF700, CD4 BV605, and CD45RO Texas Red monoclonal antibodies (BD Biosciences) then permeabilised and fixed using the FoxP3 permeabilisation solution kit (BioLegend, San Diego, CA). The fixed cells were then stained with the Ets-1 antibody directly conjugated to Alexa 647: IgG1 monoclonal antibody, 8A8 clone, (1/100, Thermo Fisher Scientific, Waltham, MA) or isotype control IgG1 (Thermo Fisher Scientific).

### Ets-1 siRNA transfection

Jurkat T cells were transfected with Ets-1 siRNAs (Santa Cruz Biotechnology, Dallas, TX) using the Neon^TM^ Transfection System Kit (Invitrogen, Carlsbad, CA) according to the manufacturer’s instructions. Control siRNA-A (Santa Cruz Biotechnology) was used as negative control. Briefly, Jurkat T cells were re-suspended in Neon Re-suspension Buffer R at a concentration of 2 × 10^7^ /mL, and each construct was transfected at a final concentration of 200 nM per 1 × 10^6^ Jurkat T cells in a 100 μl transfection volume using the Neon electroporation transfection system. Cells were cultured in 2 mL of antibiotic-free medium at a density of 5 × 10^5^ /ml in 6-well plates for 2 days followed by PMA/I stimulation. Cells were rested for three days after stimulus withdrawal and re-stimulated.

### Western blotting

Western blot analysis of Jurkat cell nuclear extracts was performed using a primary anti-rabbit antibody to Ets-1 (SC-350) and a secondary HRP-conjugated anti-rabbit antibody (AP187P; Merck Millipore). Signals were detected by enhanced chemiluminescence with film exposure. The intensity of bands of the correct molecular weight were analysed with ImageJ, and protein loading was normalised with a highly sensitive quantitative total protein loading control detection kit (Novex Reversible Membrane Protein Stain Kit, Novex, Invitrogen).

### Immunofluorescence microscopy

Cells were permeabilised by incubation with 2% Triton X-100 for 20 min. Cells were probed with primary antibodies to either NFATC1 or Rela followed by visualisation with secondary rabbit (A10042; Life Technologies), mouse (A31574; Life Technologies), goat (A11055; Life Technologies) immunoglobulins conjugated to Alexa Fluor 488, 568, or 633 nm and co-stained with DAPI. Staining was analysed by confocal laser scanning microscopy. Single 0.5 μm image stacks were obtained using a Nikon x 60 oil immersion lens on the Nikon C1 plus confocal system and the final image obtained by averaging four sequential images from the same tissue section. Digital confocal images were analysed with Fiji-ImageJ software^63^ to determine the total nuclear fluorescence intensity (TNFI) by selecting a region of interest (ROI) corresponding to the nucleus with background subtracted measured in a minimum of 20 cells for each sample set.

### RNA extraction and quantitative reverse transcription PCR (qRT-PCR)

Total RNA was extracted from Jurkat cells using TRIzol^®^ Reagent (Invitrogen) followed by chloroform extraction (Sigma) and isopropanol (Thermo Fisher Scientific) precipitation. First-strand cDNA was synthesised from 1 μg total RNA using the SuperScript^TM^ III First-Strand Synthesis System (Invitrogen). qRT-PCR was carried out to determine gene expression with gene-specific TaqMan probes in the Applied Biosystems^®^ ViiA^TM^ 7 Real-Time PCR System (Life Technologies). The sequences of all primers used in this study are listed in Table S2 in the supplemental material.

### Chromosome conformation capture (3C) assay

The 3C assay was performed according to ref. 31, except that DpnII (Genesearch, Arundel, Australia) was used as the restriction enzyme. DNA was quantified by SYBR real-time PCR with the primer sets listed in Table S2. 3C-qPCR data were normalised to a *GAPDH* loading control and primer efficiency was normalised to bacterial artificial chromosome (BAC) clones including gene deserts (GD), *DUSP10, TNFSF10*, and *BCL6* (Life Technologies).

### Chromatin immunoprecipitation (ChIP)

ChIP-PCR was performed with Magna ChIP^TM^ Protein A Magnetic Beads (Merck Millipore) as described previously^64^. ChIP-DNA enrichment was performed with antibodies targeting H2AZ, H2AZAc, H3, H3.3, H3K27ac, H3K56ac, and Ets1. ChIP-DNA was quantified by real-time PCR with the primer sets listed in Table S2 in the supplemental material.

### Formaldehyde-assisted isolation of regulatory elements (FAIRE)

FAIRE samples were prepared as outlined in ref. 65. Briefly, cells were cross-linked with 1% formaldehyde and lysed. The cell lysates were sonicated to yield an average DNA fragment distribution of approximately 200 to 500 bp. A 50 μL aliquot of fragmented DNA (total input control DNA) was reverse cross-linked at 65 °C followed by phenol-chloroform extraction. The remaining sonicated DNA (FAIRE DNA) was directly isolated by phenol-chloroform extraction and purified using the Zymo-Spin^TM^ I kit (Zymo Research). 50 ng of FAIRE DNA and the corresponding total input DNA were used to prepare the FAIRE-seq DNA library using the NEBNext^®^ Ultra DNA Library Prep Kit for Illumina^®^ (New England BioLabs Inc., Ipswich, MA) according to the manufacturer’s instructions. FAIRE DNA libraries were single-end 50 bp sequenced on an Illumina HiSeq2000 at the Ramaciotti Centre for Genomics, University of New South Wales, Sydney.

### Bioinformatics analysis

FAIRE sequencing reads were stripped of adapter sequences with CutAdapt^66^, mapped to the human (Hg19) genome with global alignment in Bowtie2^67^, and duplicate reads were removed with Picard (http://picard.sourceforge.net). Areas of enrichment in the DMSO FAIRE samples compared to the total input sample were determined using MACS2^68^ with the Broad option and using a q-value cut-off of ≤0.05. Regions from the four DMSO samples were pooled (in Galaxy) with overlapping regions merged into one. In total, 40,731 regions were detected (28 were removed because they were on chrM or chrUn). Reads were extended to 200 bp and the number of sequencing reads in each region for all samples (DMSO and rottlerin treated) were counted in R. The counts were MA normalised to each other in R. Using the NS DMSO and rottlerin treated comparison as a guide, a 1.75-fold change with a minimum read number of 30 was used as a cut-off for calling regions with changed CA. Based on the NS comparison, this was expected to give 242 regions as false positives or a 8.5–14.5% false positive rate.

The mid-points of the regions were annotated to the nearest ENSEMBL transcript using the ChIPpeakAnno package in R. As a region can be annotated to more than one transcript, regions were allocated to groups in the following order: 5′UTR, 3′UTR, exon, intron, promoter, upstream, and intergenic. A promoter was defined as the area −1 kb to 0 bp from the TSS and upstream was defined as −10 kb to −1 kb from the TSS. Regions were also annotated to RoadMap CD4^+^ memory chromatin state data^22^ and ChIP-seq data from naïve, memory, and PMA/I activated Th CD4^+^ cells donor 62 (GSE17312). Homer^69^ was used to profile the RoadMap histone marks +/−1 kb (0.1 kb bins) around the FAIRE peaks, and values were scaled by total mapped reads. Wilcoxon tests were performed with continuity correction in R. DAVID^70^ was used to look for over-represented Gene Ontology groups in the genes nearest to the accessible regions. ST >NS, SW >NS, and RS >NS regions were compared to the NS region background. A p value <0.05 and FDR <25% cut-off were used to determine significance, and groups had to have at least 10 genes present. Homer^69^ and CLOVER (with JASPAR PWM database)^71^ were used to identify over-represented transcription factor binding motifs. For both programs, the regions were standardised to 300 bp (or 500 bp for co-occupancy) and while HOMER uses genomic regions with similar GC content as background, for CLOVER NS regions were used as background. Motifs had to have a q (HOMER) or p (CLOVER) value of ≤0.05. GSEA^72^ was used to investigate the motif occurrence significance in regions differentially affected by rottlerin treatment, with sets of regions (set to 300 bp) containing JASPAR motifs (at least 2 motif occurrences, from CLOVER) instead of the usual gene lists. Regions were ranked using fold-changes, and region (‘gene’) permutation was used to establish significance. Motif co-occupancy was analysed in R using output from the CLOVER program and custom scripts (available on request). To explore the combinatory effect of different factors on chromatin accessibility outcomes, we examined every pairwise combination of the 14 enriched motifs in all regions with altered chromatin accessibility (a–n). We determined how many of the regions with both motifs belonged to a particular set as a percentage of all the sets (a-n) with altered chromatin accessibility. We also compared the composition of regions with 1 motif but not the other. Furthermore, based on the pairwise combinations, we examined several 3 motif combinations.

Genes were classified as primary response genes or memory-responsive genes if they had at least log_2_ 0.5 greater expression in ST (day 0, primary response genes) or RS cells (day 9, memory-responsive genes) compared to NS and either RS (primary response genes) or ST cells (memory-responsive genes). Expression for Jurkat cells from GSE61172^7^ and primary cells from E-MEXP-2578.

FAIRE data was deposited in GEO under accession number GSE63766.

## Additional Information

**How to cite this article**: Juan Tu, W. *et al*. Priming of transcriptional memory responses via the chromatin accessibility landscape in T cells. *Sci. Rep.* **7**, 44825; doi: 10.1038/srep44825 (2017).

**Publisher's note:** Springer Nature remains neutral with regard to jurisdictional claims in published maps and institutional affiliations.

## Supplementary Material

Supplementary Data and Information
