# Supplementary material for: Priming of transcriptional memory responses via the chromatin accessibility landscape in T cells
Source: Sci Rep. 2017 Mar 20;7:44825. doi: 10.1038/srep44825 (PMC5357947; doi:10.1038/srep44825)
Supplement: Supplementary Data and Information [file srep44825-s1.pdf]

Additional Information for:

Priming of transcriptional memory responses via the chromatin accessibility landscape in T cells

Wen Juan Tu[#1], Kristine Hardy[#1], Christopher R. Sutton[1], Robert McCuaig[1], Jasmine Li[2], Jenny Dunn[1], Abel Tan[1], Vedran Brezar[3], Melanie Morris[1], Gareth Denyer[4], Sau Kuen Lee[5], Stephen J. Turner[2], Nabila Seddiki[3], Corey Smith[5], Rajiv Khanna[5] and Sudha Rao[*1]

Tu-SFig 1

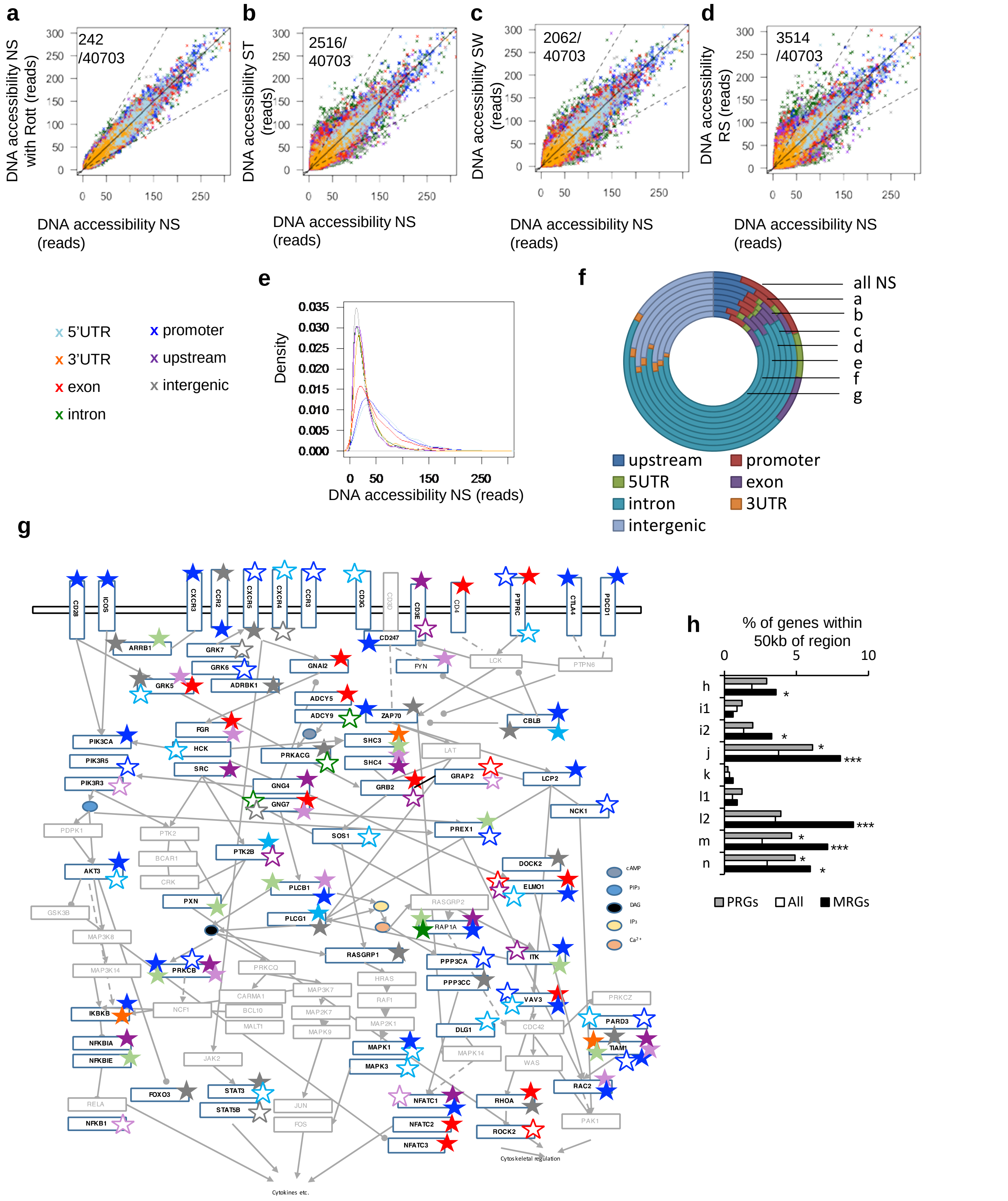

a
b
c
d
e
f
g
h
242 /40703
2516/ 40703
2062/ 40703
3514 /40703
DNA accessibility NS with Rott (reads)
DNA accessibility ST (reads)
DNA accessibility SW (reads)
DNA accessibility RS (reads)
DNA accessibility NS (reads)
5'UTR
3'UTR
exon
intron
promoter
upstream
intergenic
Density
all NS
upstream
promoter
5UTR
exon
intron
3UTR
intergenic
% of genes within 50kb of region
PRGs
All
MRGs
Cytoskeletal regulation
Cytokines etc.

Supplementary Figure 1: Regions with changes in DNA accessibility are mainly in non-promoter regions and are near genes involved in the immune response.

(**a-e**). FAIRE-seq was performed on DMSO- or rottlerin-treated Jurkat T cells. Regions of enrichment were called against total genomic input and read counts for the resulting 40,703 regions were MA normalised. Regions were considered to increase in accessibility in a given stimulation state if they had over 30 reads in that treatment and more than a 1.75-fold increase compared to NS. The density distributions for the read counts in the NS sample are also shown. Regions were annotated with respect to their nearest ENSEMBL gene. As a region can be annotated to more than one transcript, regions were allocated to groups in the following order: 5'UTR (light blue), 3'UTR (orange), exon (red), intron (green), promoter (dark blue), upstream (purple), intergenic (grey). A promoter was defined as the area -1kb to 0 bp from the TSS and upstream was defined as -10kb to -1kb from the TSS. Dotted lines show 1.75-fold changes. Numbers indicate fraction of regions with increased accessibility.

(**f**) The DNA accessible regions in the different sets and all accessible region in NS cells were annotated with respect to their genomic location.

(g) TCR and chemokine KEGG signalling pathways showing genes near regions with increased (filled star) or decreased (empty star) accessibility. Colours of stars correspond to Venn diagram groups (Fig. 1 c, d).

(**h**) The percentage of primary response genes (PRGs), all genes on the array, and memory responsive genes (MRGs) with a TSS within 50kb of a region for the different subsets. Genes were classified as PRGs or MRGs if they had at least $\log_2$ 0.5 greater expression in ST (day 0, PRGs) or RS cells (day 9, MRGs) compared to NS and either RS (PRGs) or ST cells (MRGs). Expression from GEO: GSE61172. *$p< 0.05$, ** $p< 0.001$, ***$p < 1x10^{-5}$, compared to all genes on array, Fisher's exact test.

Tu-SFig 2

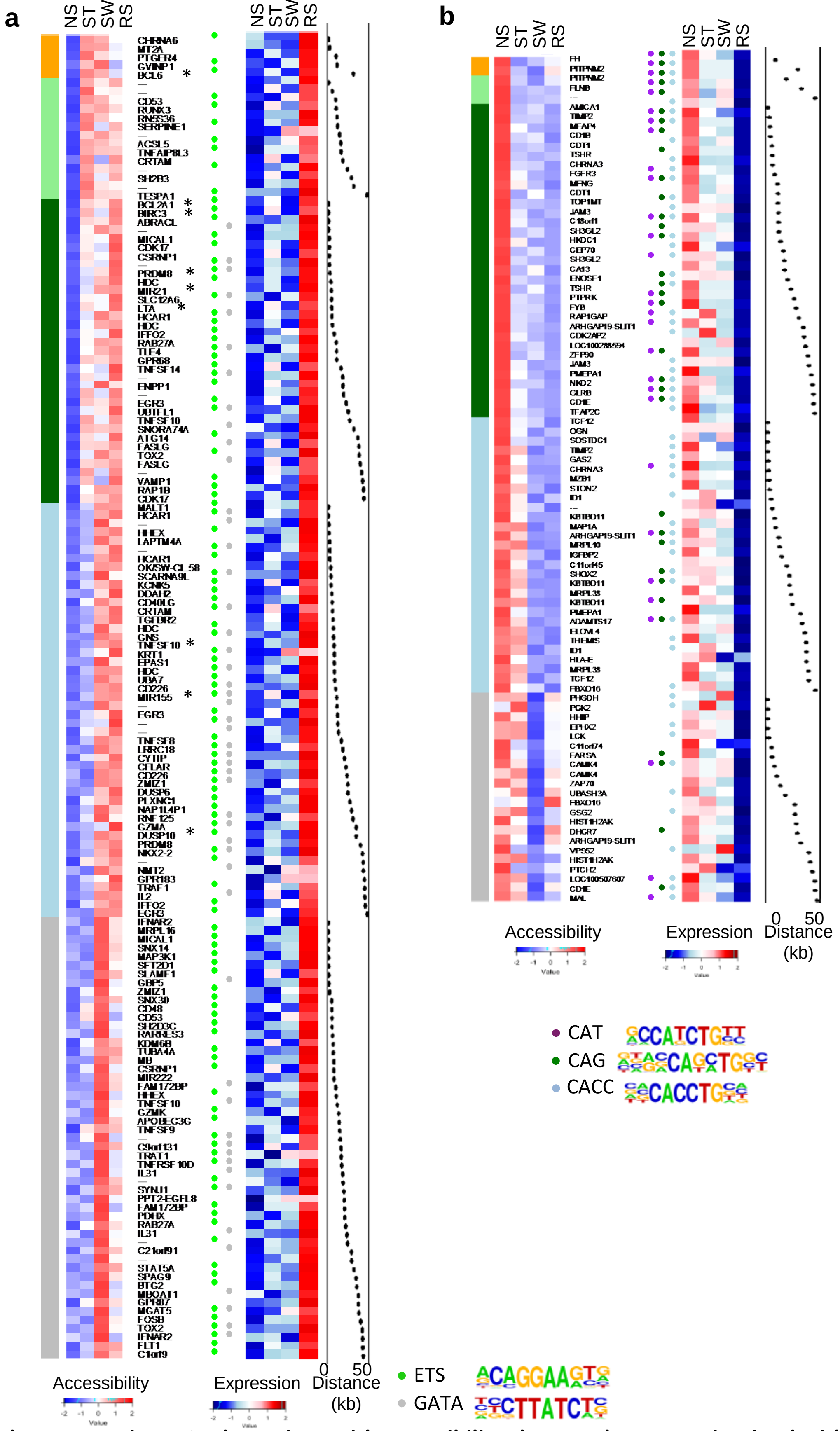

Supplementary Figure 2: The regions with accessibility changes that are maintained with stimulus withdrawal and are near memory response genes.

(**a**) All regions with increased accessibility maintained on stimulus withdrawal and that are within 50kb of the TSS of a memory responsive gene (MRG). * denotes regions chosen for further analysis. Regions with ETS or GATA DNA binding motifs are annotated.

(**b**) All regions with decreased accessibility maintained on stimulus withdrawal and that are within 50kb of the TSS of a gene with lower expression upon secondary stimulation. Regions with the different bHLH DNA binding motifs are annotated.

(**a-b**) Heat maps show Z-scaled values for accessibility of the region and expression of the gene. The distance between an accessible region and the TSS of the gene is shown. The side colour indicates which set the regions belong to.

Tu-SFig 3

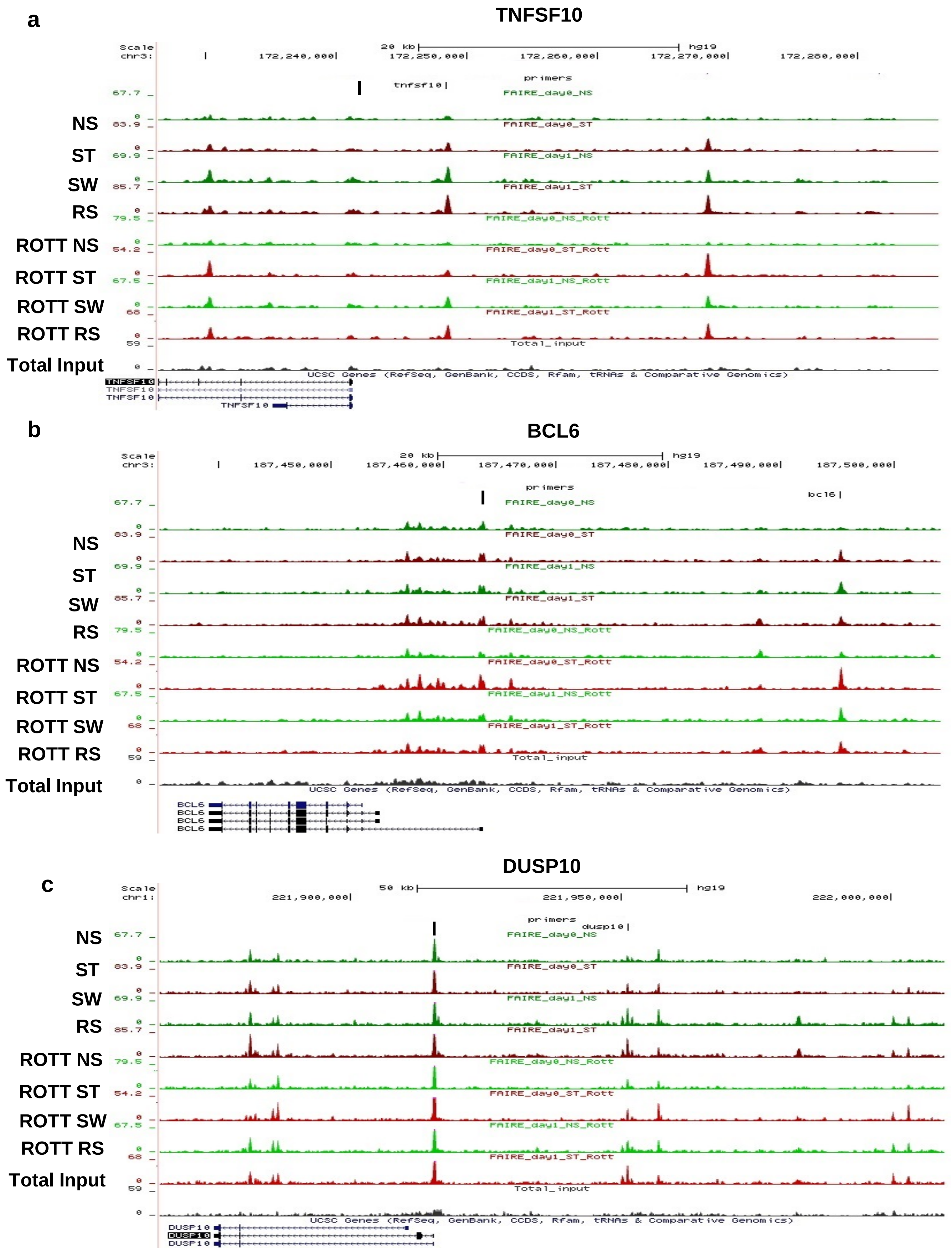

Supplementary Figure 3: Accessible regions near the memory responsive genes *TNFSF10, BCL6*, and *DUSP10*.
(a) - (c) Chromatin accessibility by FAIRE-seq across *TNFSF10*, *BCL6,* and *DUSP10* genes in DMSO or rottlerin (ROTT) treated NS, ST, SW, and RS cells with the total input sample. Data are shown in UCSC Genome Browser Hg19. The location of FAIRE-qPCR and 3C-qPCR primers are indicated.

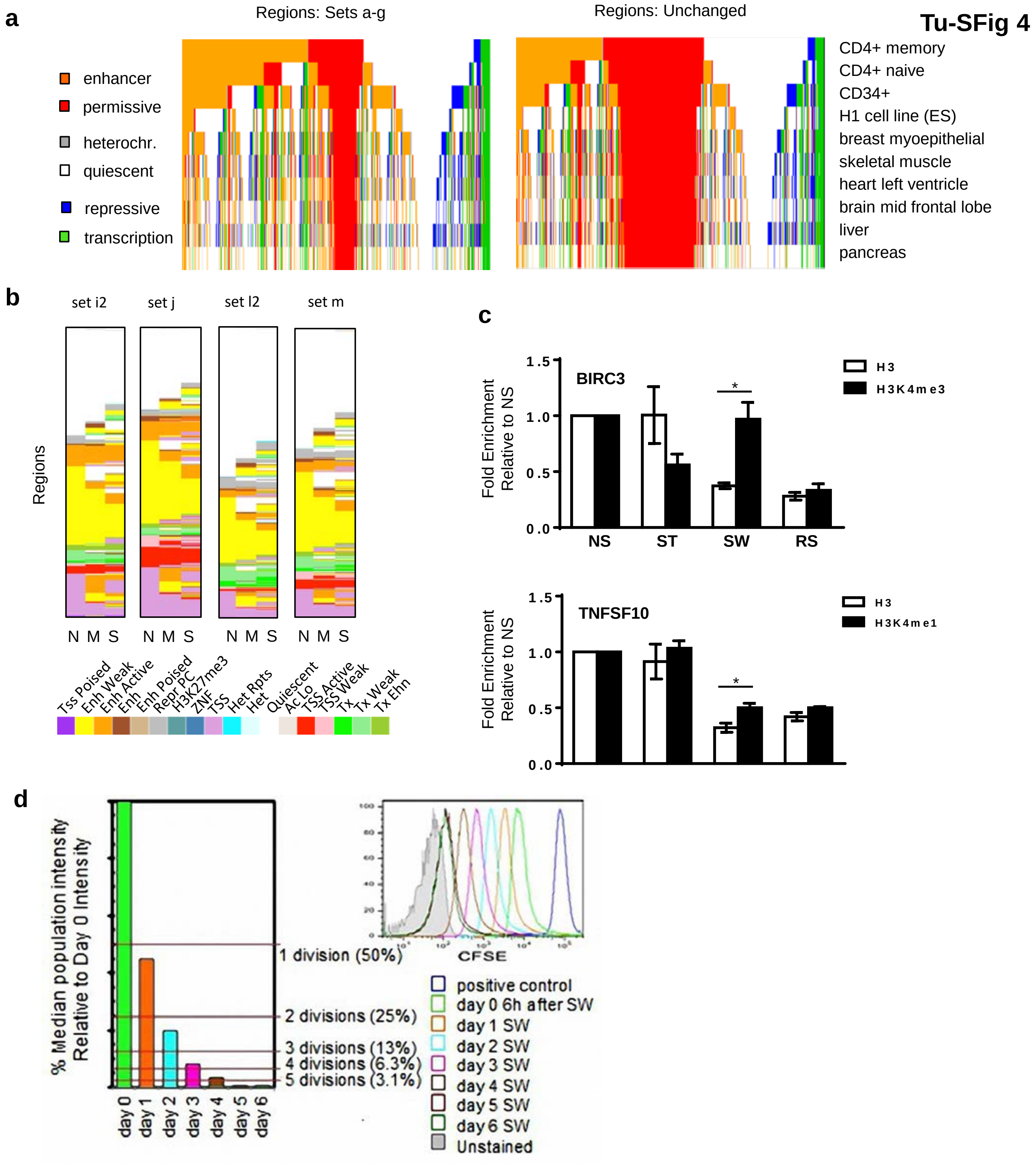

Supplementary Figure 4: The chromatin environment of the regions with altered accessibility.

(**a**) Regions in all the sets with increased accessibility, different sets with increased accessibility, or regions with unchanged accessibility were annotated with respect to their chromatin state segmentation in different cell types using Roadmap data. Quiescent (quies, low level of any marks), repressive (repr, H3K27me3), transcription (trans, H3K36me3), enhancer (enh, H3K4me1), permissive (perm, H3K4me3), and heterochromatin (het, H3K9me3).

(**b**) Detailed chromatin state of the regions with decreased accessibility associated with transcriptional memory in naïve (N), memory (M), and PMA/I stimulated Th (S) CD4 lymphocytes. Transcribed (Tx), Permissive (Tss), Enhancer (Enh).

(**c**) Fold change in ChIP-qPCR analysis of H3 and H3K4me3 binding at the *BIRC3* promoter and H3 and H3K4me1 binding at the *TNFSF10* enhancer in the Jurkat TM model. ChIP enrichment ratio relative to NS is shown (mean ± SEM, n=3 biological replicates, * $p < 0.05$, t-test).

(**d**) Cell division after the initial stimulation with PMA and ionomycin. The intensity of carboxyfluroescein succinimidyl ester (CFSE) was assessed at 6 h and day 1-6 after SW. The median intensity of CFSE is shown after subtraction of the unstained cell intensity and as a percentage of the day 0 SW sample. A 2-fold decrease in CFSE intensity indicates one cell division. A representative of 2 biological experiments.

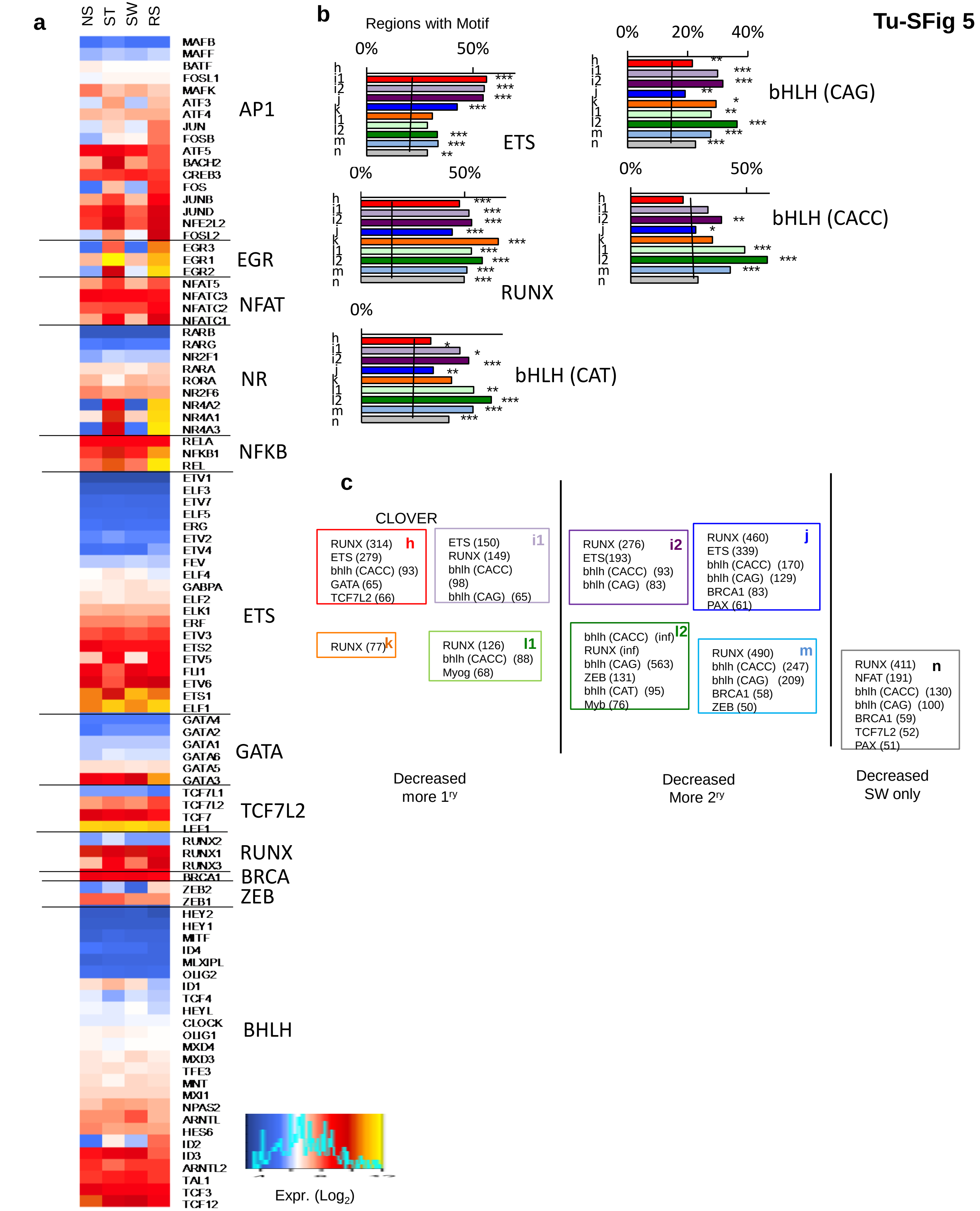

Supplementary Figure 5: The transcription factor motifs that characterise the different sets of decreased accessibility.

(**a**) Expression levels of the transcription factors that bind the over-represented motif families. Expression scale is log2. Levels in Jurkat TM model from GEO: GSE61172.

(**b**) The top over-represented motifs in the region sets with the percentages of the regions containing the motifs. Results from HOMER, *p< 0.05, ***p < 1x10^{-5}, Benjamini q value, compared to control sets with similar GC content (trend line).

(**c**) The top over-represented JASPAR motif families for the different sets with decreased accessibility. Enrichment scores > 50 (scores shown in brackets) and CLOVER significance p value < 0.05 relative to NS regions.

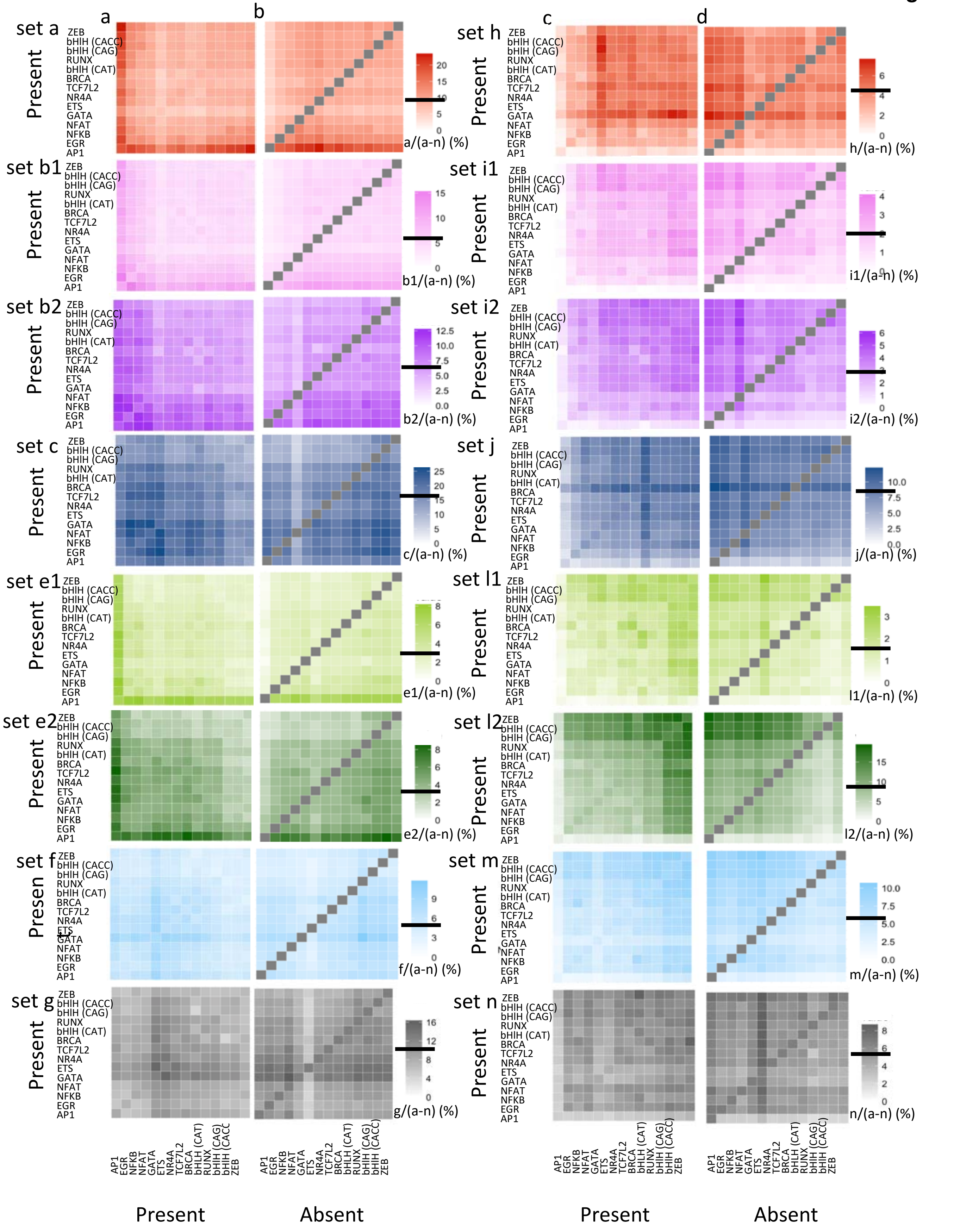

Supplementary Figure 6: The influence of different combinations of DNA binding motifs on the accessibility profile. The effect of different motif combinations on the relative proportions of regions in each accessibility set. **A, C**: Plots show the percentage of regions with both motifs that are in the given set compared to all altered (a-n) regions containing those two motifs. **B, D**: Plots show the percentage of regions containing the 'Present' motif but not the 'Absent' motif that are in the given set compared to all altered (a-n) regions with those criteria. Black line on scale indicates the percentage of the set without motif restrictions. Regions were extended to 500bp.

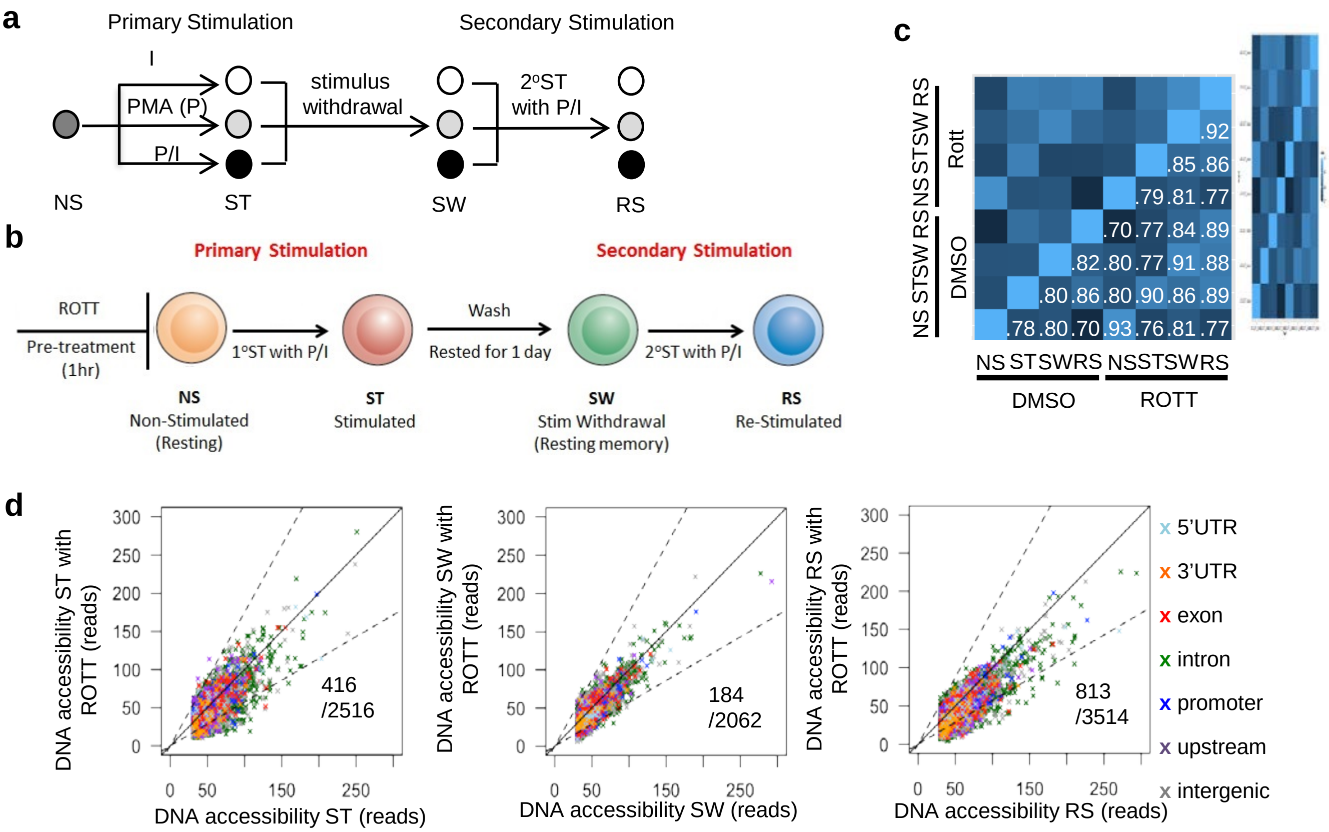

Supplementary Figure 7: The effect of rottlerin on changes in DNA accessibility.

(**a**) A schematic of the experimental design. Non-stimulated (NS) Jurkat T cells stimulated with ionomycin alone (I), PMA alone (PMA), or PMA and ionomycin (P/I) were subjected to stimulus withdrawal and rested for 1 day. These cells were then reactivated with full strength P/I.

(**b**) A schematic of the experimental design for examining the effect of rottlerin (ROTT) pre-treatment on DNA accessibility in the Jurkat TM model. The ROTT was washed away with the stimulus withdrawal and not added upon re-stimulation.

(**c**) Spearman correlations of the FAIRE-seq samples.

(**d**) The effect of rottlerin on regions with increased accessibility (compared to NS) in ST SW and RS is shown. Regions were considered to increase in accessibility in a given treatment if they had over 30 reads in that treatment and more than a 1.75-fold increase compared to NS. Regions were considered rottlerin sensitive if they had a 1.75-fold decrease in the Rott sample for the matching treatment. Regions were annotated with respect to their nearest ENSEMBL gene. As a region can be annotated to more than one transcript, regions were allocated to groups in the following order: 5'UTR (light blue), 3'UTR (orange), exon (red), intron (green), promoter (dark blue), upstream (purple), intergenic (grey). A promoter was defined as the area -1kb to 0 bp from the TSS and upstream was defined as -10kb to -1kb from the TSS. Dotted lines show 1.75-fold changes. The fraction of regions with decreased accessibility in the presence of rottlerin are indicated.

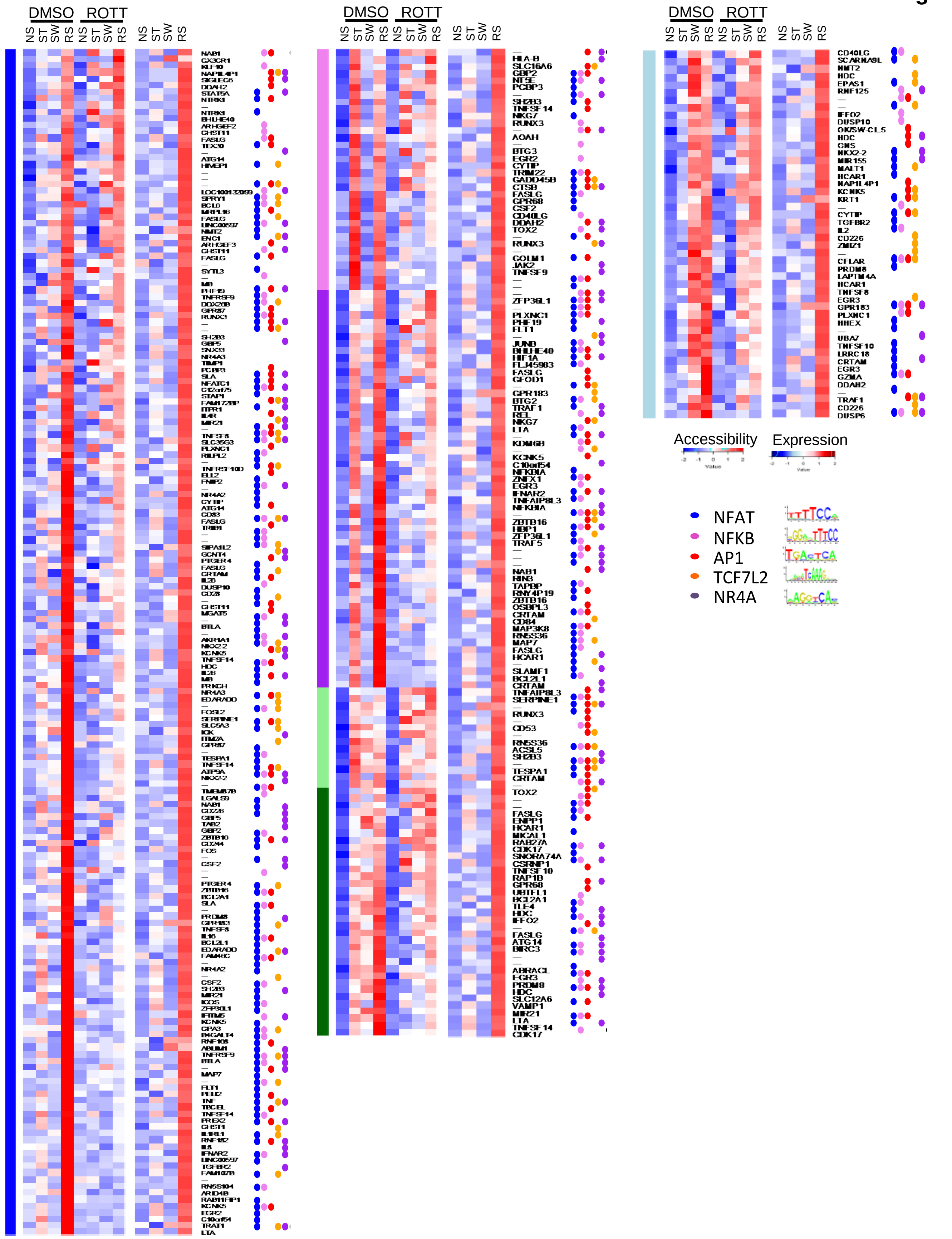

Supplementary Figure 8: The regions with increased accessibility in re-stimulated cells that are close to MRGs. All regions with increased accessibility in re-stimulated cells that are within 50kb of the transcription start site of a memory responsive gene (MRG). The side colour indicates which set the regions belong to, and, within sets, the regions are ranked by the inhibitory effect of rottlerin (ROTT) pre-treatment in the RS cells. Regions with NFAT, NF-κB, AP1, TCF7L2, or NR4A DNA binding motifs are annotated. Heat maps show Z-scaled values for accessibility of the region and expression of the gene.

a

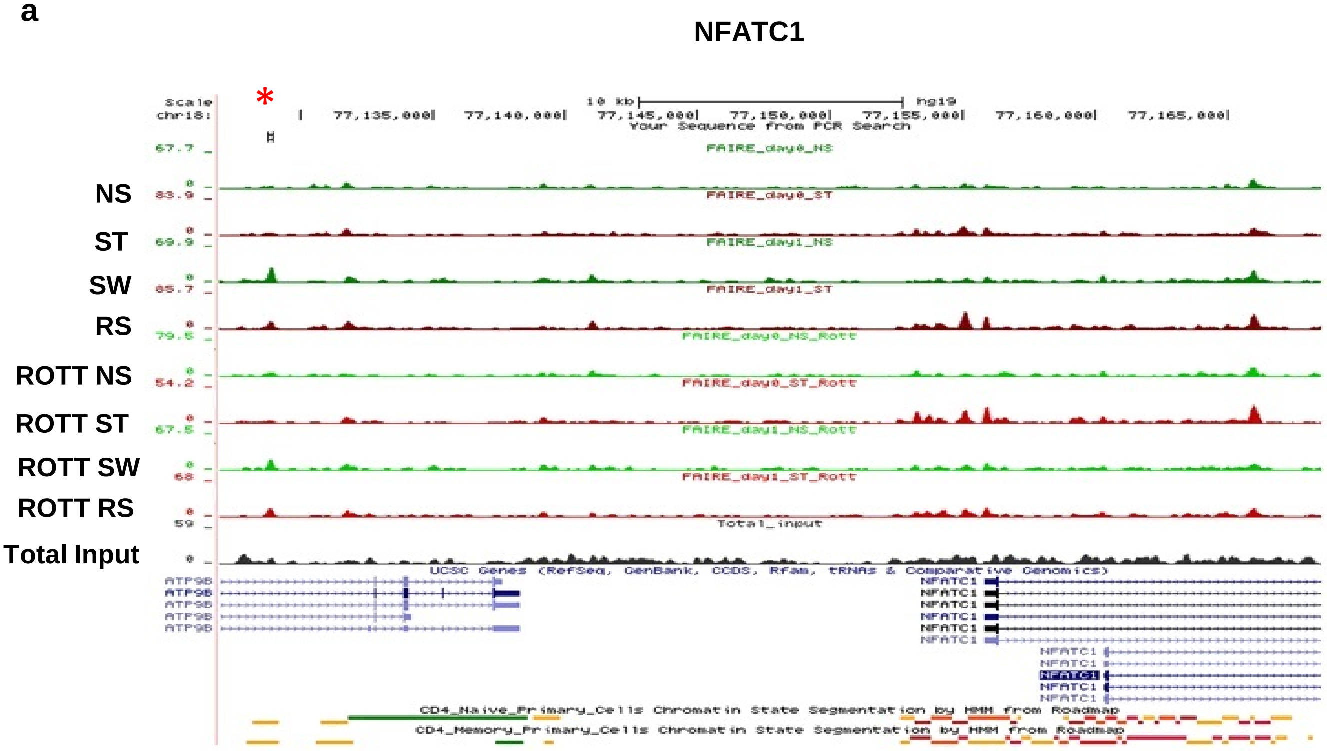

b

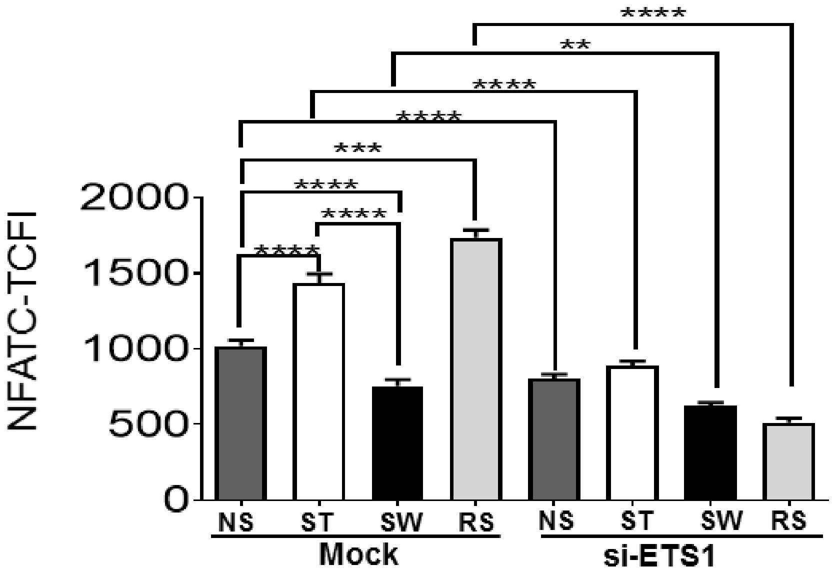

Supplementary Figure 9: Accessible regions near *NFATC1*.

(a) Chromatin accessibility by FAIRE-seq near the *NFATC1* gene in DMSO- or rottlerin-(ROTT) treated Jurkat NS, ST, SW, and RS cells with the total input sample. Data are shown in UCSC Genome Browser Hg19. The Roadmap annotation in $CD4^+$ Naïve and memory lymphocytes is also shown. The location of FAIRE-qPCR is indicated by a red *. (**b**) The cytoplasmic levels of NFATC1 in P/I stimulated (ST), SW, and re-stimulated (RS) Jurkat cells pre-treated with mock or ETS1 siRNA. SW cells were rested for 3 days. Total cytoplasmic fluorescence intensity (TCFI) from 3 biological replicates with n = 20 cells each mean ± SEM. * $p < 0.05$, ** $p < 0.01$, *** $p < 0.001$, **** $p < 0.0001$. Mann–Whitney test.

Supplementary Table 1: Motifs enriched in regions with greater accessibility in cells cultured under different conditions (ST, SW and RS) compared to NS, when these regions are ranked by their accessibility in the presence of rottlerin.

ST>NS

NAME	Jaspar motif	SIZE	ES	NES	NOM p-val	FDR q-val	FWER p-val
MA0152.1	>MA0152.1 NFATC2	361	0.42231718	2.4128845	0	0	0
MA0107.1	>MA0107.1 RELA	196	0.3618986	1.967459	0	0.01004726	0.021
MA0105.3	>MA0105.3 NFKB1	129	0.36520037	1.9499753	0	0.0070204	0.022
MA0463.1	>MA0463.1 Bcl6	43	0.4117741	1.8352141	0.00102354	0.01530886	0.062
MA0520.1	>MA0520.1 Stat6	107	0.34989223	1.7998922	0.002002	0.0168237	0.083
MA0101.1	>MA0101.1 REL	218	0.32739332	1.7973495	0	0.01401975	0.083
MA0037.2	>MA0037.2 GATA3	61	0.3530035	1.6901157	0.00303951	0.03035266	0.2
MA0088.1	>MA0088.1 znf143	65	0.34004283	1.6417781	0.00705645	0.04032629	0.292

SW>NS

NAME	Jaspar motif	SIZE	ES	NES	NOM p-val	FDR q-val	FWER p-val
MA0523.1	>MA0523.1 TCF7L2	98	0.38324198	2.3038177	0	0.00274737	0.002
MA0037.2	>MA0037.2 GATA3	127	0.2846466	1.7899082	0	0.10021869	0.203
MA0036.2	>MA0036.2 GATA2	88	0.2798465	1.6871287	0.0040282	0.15834786	0.417

RS>NS

NAME	Jaspar motif	SIZE	ES	NES	NOM p-val	FDR q-val	FWER p-val
MA0152.1	>MA0152.1 NFATC2	737	0.40947014	2.4211063	0	0	0
MA0107.1	>MA0107.1 RELA	284	0.32737404	1.8430357	0	0.01266953	0.027
MA0105.3	>MA0105.3 NFKB1	181	0.32138032	1.7417564	0	0.02219816	0.068
MA0101.1	>MA0101.1 REL	302	0.28150713	1.5781208	0	0.09794883	0.347
MA0523.1	>MA0523.1 TCF7L2	167	0.2841619	1.5487168	0.002	0.10141807	0.435

Supplementary Table 2: Oligos and Taqman probes used in q-PCR, CHIP-PCR and 3C.

Primer	Sequence
**FAIRE and ChIP**	
Bcl2a1_PROM_F	TGGACGTTTTGCTTGGACCT
Bcl2a1_PROM_R	AAGGTGAGCCAGCTCAAGAC
mir155_PROM_F	ATTGGCAGGGTTAGGTGGTG
mir155_PROM_R	ACAGAAATCAGGGAGGCAGC
BIRC3_PROM_F	TTGGGTCATGGAAATCCCCG
BIRC3_PROM_R	CCCCACCCCTATCTGTACCA
TNF_enh_F	TCCAGTCATCAGCTCTCCCA
TNF_enh_R	GCGAAGGATGCTCCTTGAGA
BCL6_enh_F	GGACAGGTCTTCCACTCCAG
BCL6_enh_R	GCATTGAGGTGGCAGAAACC
TNFSF10_enh_F	TCAGTGCCTTCTTCTGCTTCA
TNFSF10_enh_R	AGCTAGGGTGTGACATGACC
PRDM8_enh_F	GAGGAAGAGACAGGGATGGC
PRDM8_enh_R	GGTGGTTTCCTGTGTCAGCA
DUSP10_enh_F	TAGATGTGTGGGAGCAGGGA
DUSP10_enh_R	CTGCAAGGGGAGGGAATTGT
MIR21_FAIRE_F	TTGGATAAGGATGACGCACA
MIR21_FAIRE_R	TCAGAAGTCCCACATTTATCACC
DUSP2_enh_F	GGGATGGAGGTGAGAGTGAC
DUSP2_enh_R	CATCGGTGTGTTCGTCAGAG
TRIB3_prom_F	GAGTCCGTGGCTGATGTCT
TRIB3_prom_R	CCTCTCTCCTCTGCATCCG
BACH2_prom_F	GAACGCCCACACTCTCAATC
BACH2_prom_R	AAGTTATTGTGAATGGGGAGCG
CREB5_prom_F	CCTCCGTGGTCTTGTCTGAA
CREB5_prom_R	GACACGGCTCCCTGACATTA
PPIA_prom_F	GCCAGGCTCCTGTTTTAATG
PPIA_prom_R	GAGCAGTCTCCGGTTTTGAG
GAPDH_prom_F	AGTCCAGTCCTGGGAACCA
GAPDH_prom_R	TAGTAGCCGGGCCCTACTTT
IL2_prom_F	CTTGCTCTTGTCCACCACAA
IL2_prom_R	ACCCCCAAAGACTGACTGAA
TNF_prom_F	GCTTGTGTGCCCCAACTTT
TNF_prom_R	TGTGCCAACAACTGCCTTT
NFATC1_enh_F	CAGCATACTCCGTCGTTGG
NFATC1_enh_R	CAATTAACTGGGGCTCTGC
ID2_enh_F	TCCGATGGTATCTCGTCCC
ID2_enh_R	AACCTCGAGCTTTCTACACC
ICOS_enhU_F	TTTTTGAGAAGGCGAGGCAT
ICOS_enhU_R	TGCTTTTTCCCAACCCTCTC
IL8_enh_F	CAGTCTTCAACAGAGGAAAGAGC
IL8_enh_R	TCCATTCAGTCTGTTTCAGCTAC
FASLG_prom_F	GGTTTGCTCTGAGCTTCTTG
FASLG_prom_R	GGCAAGCTGGATCTCTCTTA
**3C**	
GD_anchor	AGATTTCTGACTCTAAAAAGTCCTTGGCTGTACTG
GD53*	CTTTGTGGAACAGCCATTGGCAAAAGTCC
GD55*	CAGTCAAACTTAGATACGCAGAGGCAAGG
GD58*	CATCTCACTGCCATAAAGCACTTCAGAATGG
DUSP10_anchor	ACACTCACACAAGCACCGCCTTACAATC
DUSP10_Reg	GAAATTGCCAGTACCTAGTGAGCTAATGACC
BCL6_anchor	CTGTAGCAAAGCTCGGCCTCTGGAATTC
BCL6_Reg	TTAACACAGTGCTTCACTGCCTCTATCAAGTC
TNFSF10_anchor	ACCCACATCTATTGAACCTGCAACTGTC
TNFSF10_Reg	AGACAAACACTGGGTAGTCCAAGGAGGATG
**mRNA**	
**Gene**	**Taqman probe ID**
IL2	Hs00174114
TNF	Hs00174128
GAPDH	Hs99999905
BIRC3	Hs00985031_g1
mir21	Hs00978580_m1
mir155	Hs01374569_m1
BCL2a1	Hs00187845_m1
TNFSF10	Hs00921974_m1
DUSP10	Hs00200527_m1
BCL6	Hs00153368_m1
PRDM8	Hs01027637_m1
PPIA	Hs04194521_s1
Ets1	Hs00428293_m1
